# Structural and functional insight into the regulation of kinesin-1 by microtubule associated protein MAP7

**DOI:** 10.1126/science.abf6154

**Published:** 2022-01-20

**Authors:** Luke S Ferro, Qianglin Fang, Lisa Eshun-Wilson, Jonathan Fernandes, Amanda Jack, Daniel P Farrell, Mert Golcuk, Teun Huijben, Katelyn Costa, Mert Gur, Frank DiMaio, Eva Nogales, Ahmet Yildiz

**Affiliations:** 1 Department of Molecular and Cellular Biology, University of California, Berkeley CA, USA; 2 Department of Chemistry, University of California, Berkeley CA, USA; 3 Biophysics Graduate Group, University of California, Berkeley CA, USA; 4 Department of Biochemistry, University of Washington, Seattle, WA, USA; 5 Department of Mechanical Engineering, Istanbul Technical University, Istanbul, Turkey; 6 Department of Imaging Physics, Delft University of Technology, Delft, Netherlands; 7 Press West Illustrations, Boston MA, USA; 8 Howard Hughes Medical Institute, Chevy Chase MD, USA; 9 Physics Department, University of California, Berkeley CA, USA

## Abstract

The microtubule (MT)-associated protein, MAP7 is a required cofactor for kinesin-1 driven transport of intracellular cargoes. Using cryo-electron microscopy and single-molecule imaging, we investigated how MAP7 binds MTs and facilitates kinesin-1 motility. The MT-binding domain (MTBD) of MAP7 bound MTs as an extended α-helix between the protofilament ridge and the site of lateral contact. Unexpectedly, the MTBD partially overlapped with kinesin-1’s binding site and inhibited kinesin-1 motility. However, by tethering kinesin-1 to the MT, the projection domain of MAP7 prevented dissociation of the motor and facilitated its binding to available neighboring sites. The inhibitory effect of the MTBD dominated as MTs became saturated with MAP7. Our results reveal biphasic regulation of kinesin-1 by MAP7 in the context of their competitive binding to MTs.

## One Sentence Summary

MAP7 binds to microtubules as an extended α-helix and activates kinesin-1 motility despite the overlap between their tubulin binding sites.

Kinesin and dynein are microtubule-associated molecular motors that are differentially regulated to deliver intracellular cargos to their destinations (1, 2). Intracellular cargos are sorted by structural MAPs that decorate the MT surface (1). Distinct cellular localizations of MAPs correlate with their regulatory roles in intracellular traffic (3). Overexpression of tau disrupts kinesin-1 (kinesin hereafter) transport of synaptic vesicles in axons (4, 5), while the knockdown of tau rescues defects in axonal transport in Alzheimer’s disease models (6). Unlike tau, MAP7 is a required cofactor for kinesin-driven transport in cells (7–9). The MAP7 projection domain binds to kinesin’s coiled-coil stalk in vitro (9, 10), recruits kinesin to MTs, and activates its motility (9, 11). Transient interactions with the MAP7 projection domain may enable kinesin to hop from one MAP to another, increasing its apparent run length by disfavoring detachment from the MT (9).

**Fig. 1.**
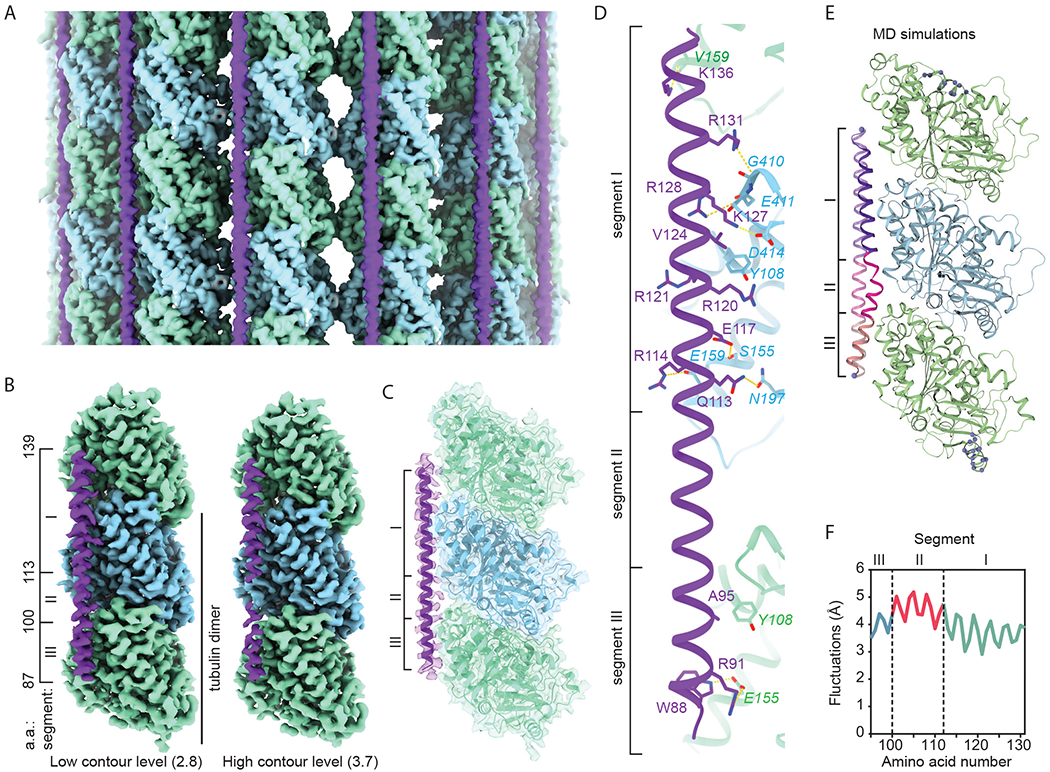
MAP7 binds the MT between the outer protofilament ridge and the site of lateral contacts. **(A)** Cryo-EM map (without symmetry expansion) of an MT decorated with MAP7; α-tubulin, β-tubulin, and MAP7 are shown in green, blue, and purple, respectively. **(B)** Improved MAP7-MT cryo-EM map after symmetry expansion and protofilament-based density subtraction (see Methods). MAP7 binds across both inter- and intra-dimer interfaces, although weaker density is seen for the region over the intra-dimer interface (segment II), indicative of more flexibility and weaker interaction. Only one repeat of MAP7 and its neighboring tubulins is shown for clarity. **(C)** Ribbon diagram for MAP7 and tubulin with the improved cryo-EM density map shown in gray. **(D)** Details of the interacting residues between MAP7 and its neighboring tubulins. **(E)** Initial and final MAP7-tubulin conformations obtained from an example all-atom MD simulation. Beads represent constrained atoms in MAP7 and tubulin. **(F)** The average root-mean-squared fluctuations of MAP7 Cα atoms from the cryo-EM structure coordinates within 200 ns (*n* = 4 simulations).

To understand how MAP7 regulates kinesin, we determined the cryo-electron microscopy (cryo-EM) structure of MTs decorated with full-length (FL) MAP7 (Fig. 1A–B, fig. S1A, Tables S1–2, and movie S1). The reconstruction revealed a 53 residue-long α-helix that runs parallel to the MT axis about the length of a tubulin dimer (Fig. 1A–C, fig. S1B). Unlike MAP2, MAP4, and tau, which bind along the outer ridges of the protofilaments (12–14), MAP7 ran halfway between the outer ridge and the site of lateral contacts. The co-structure of MAP7’s MT binding domain (MTBD; residues 60-170) and FL tau on the MT (fig. S2) illustrated their distinct MT footprints and confirmed that the helical segment corresponds to the MAP7’s MTBD. We identified a single MAP7 sequence register that corresponds to a well conserved segment of the MTBD (residues 87-139; fig. S3A–C) via Rosetta modeling (15), and validated this registry by determining the structure of MTs decorated with a shorter MAP7 construct (residues 83-134; fig. S3D). Because MAP7 MTBD could potentially form a helix longer than the length of a tubulin heterodimer (fig. S2A), we cannot exclude the possibility of a larger footprint of MAP7 on the MT ((13), fig. S4 and Methods).

The α-helical density for MAP7 is not uniform (Fig. 1B). Segment I (residues 113-139), which is the best-resolved region (Fig. 1B), makes extensive interactions with tubulin (Fig. 1D): Q113 and E117 of MAP7 are within hydrogen-bonding distance of N197 and S155 of β-tubulin, respectively; R114 and K127 of MAP7 engage in electrostatic interactions with E159 and D414 of β-tubulin, respectively. Y108 of β-tubulin inserts into a hydrophobic pocket formed by R120, R121, and V124 of MAP7. We also identified potential hydrogen bonds between R128, R131, and K136 of MAP7 and the mainchain oxygens of E411 and G410 of β-tubulin and V159 of α-tubulin, respectively. Segment III (residues 87-99) interacts with α-tubulin. Segment II (residues 100-113) faces a cavity at the intra-tubulin dimer and has the weakest density in our map (Fig. 1B). All-atom molecular dynamics (MD) simulations verified these pairwise interactions, and additional potential interactions between MAP7 and tubulin (fig. S5, movie S2). Segment II fluctuated more than other segments in simulations because it only makes transient contacts with tubulin (Fig. 1E–F, fig. S5).

**Fig. 2.**
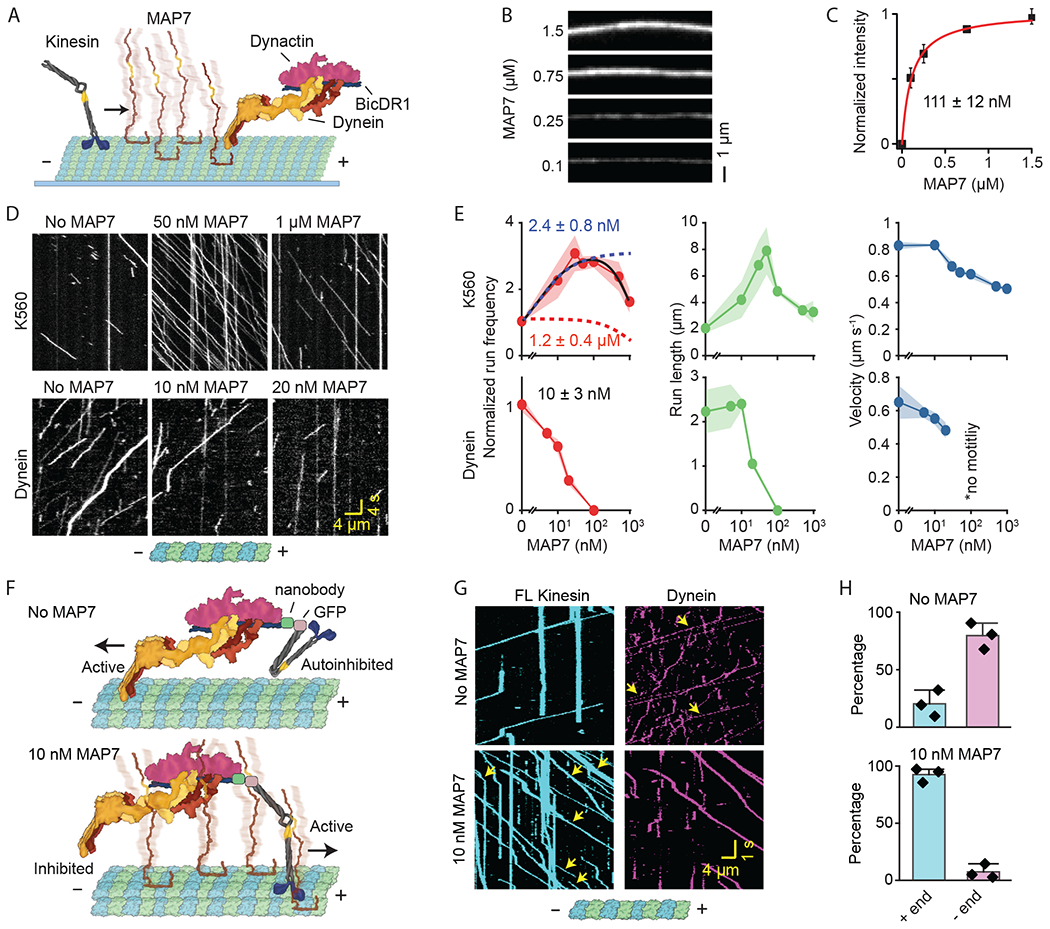
MAP7 differentially regulates kinesin and dynein motility. **(A)** Schematic of kinesin and dynein motility on MTs coated with MAPs. **(B)** MT decoration of fluorescently-labeled MAP7 under different concentrations (μM). **(C)** Fluorescence intensity (mean ± s.d.) of MAP7 was fit to the Hill equation (solid curve) to calculate K_D_ (±s.e.). From left to right, N = 44, 50, 68, 109 MTs for MAP7 (two technical replicates). **(D)** Kymographs of K560 and DDR in the presence of MAP7. Assays were performed in 150 mM KAc and 0.1% methylcellulose. **(E)** Run frequency, run length, and velocity of K560 and DDR at different MAP7 concentrations (mean ±s.e.m.). K560 run frequency was fit to biphasic Hill equation (solid black curve) to reveal the half-maximal activation (blue dashed curve) and inhibition (IC_50_, red dashed curve) concentrations (±s.e.). DDR run frequency was fit to Langmuir equation (not shown) to calculate IC_50_ (± s.e.). From left to right, *n* = 281, 463, 532, 836, 381, 433, 233 for K560, and 386, 235, 213, 146 for DDR; two technical replicates). **(F)** Schematic of kinesin and dynein assembled onto BicDR1. The addition of MAP7 switches the active motor. **(G)** Kymographs of kinesin-dynein assemblies with and without 10 nM MAP7. Yellow arrows show kinesin-dynein colocalizers. **(H)** The directionality of kinesin-dynein assemblies (mean ±s.d., *n* = 356 and 580 for 0 and 10 nM MAP7, respectively, three replicates).

We next determined how MAP7 affects the motility of kinesin and the dynein-dynactin-BicDR1 complex (DDR, dynein hereafter) (16) (Fig. 2A). FL MAP7 uniformly decorated MTs with a dissociation constant of 111 ± 12 nM (±s.e.) under physiological salt (150 mM KAc, Fig. 2B–C, fig. S6). As previously reported (9, 11), the addition of 50 nM MAP7 rescued FL kinesin from autoinhibition (17) and substantially increased its run frequency and length (fig. S7). MAP7 also enhanced the motility of constitutively-active kinesin (K560, residues 1-560) (Fig. 2D–E, movie S3) (9, 11). Unlike run length and frequency, kinesin velocity decreases even at low MAP7 concentrations (Fig. 2E), which could be because of pausing at MTBD obstacles or binding to the projection domain. Although dynein has been reported not to be inhibited by 5 nM MAP7 (10), we found that it was inhibited by MAP7 with half-max inhibition constant of 10 ± 3 nM (Fig. 2D–E). MAP7 decoration of MTs also switched the direction of an assembly that links FL kinesin to dynein (Fig. 2F) (18). While 80% of kinesin-dynein assemblies were minus-end-directed on undecorated MTs, 93 % moved towards the plus-end in 10 nM MAP7 (Fig. 2G–H, fig. S6C, movie S4).

**Fig. 3.**
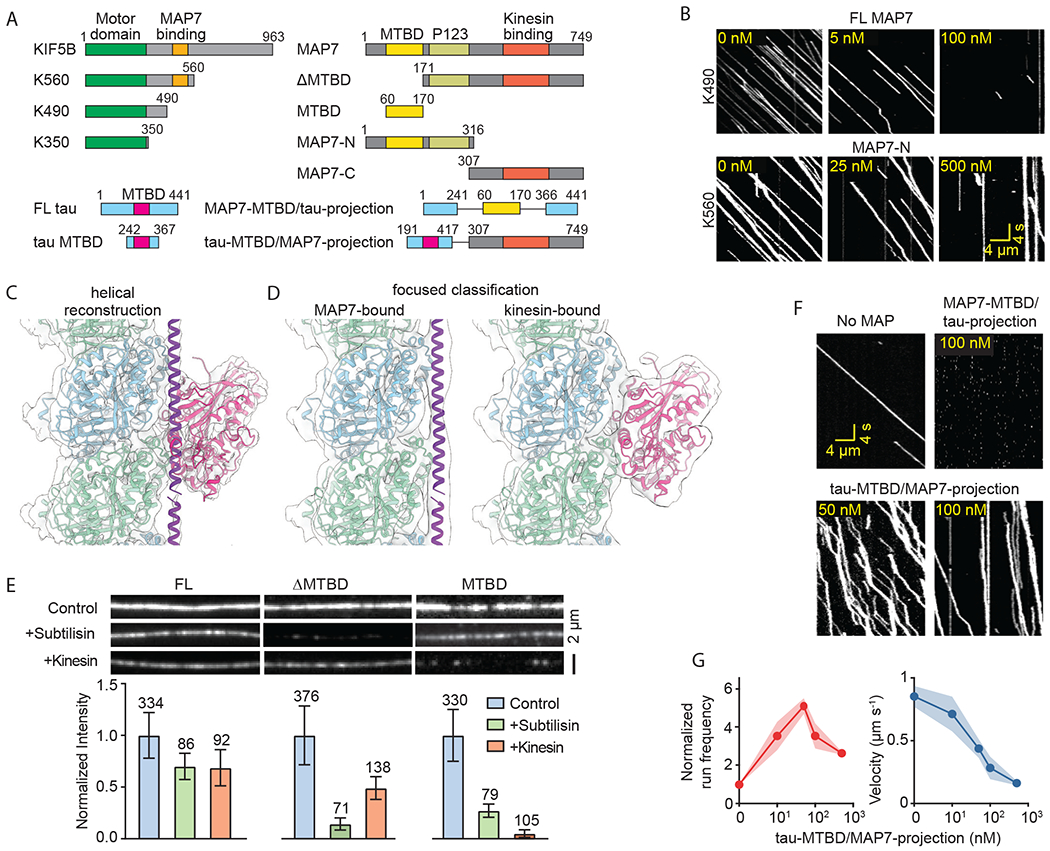
MT tethering enables kinesin to move on MAP7-decorated MTs despite their overlapping binding sites. **(A)** Schematic of kinesin, MAP7, and tau constructs. **(B)** Kymographs show kinesin motility in the presence of FL and truncated MAP7. **(C)** Average cryo-EM map of an MT decorated with both MAP7 and rigor kinesin. Atomic model of MAP7 and previously reported structure of kinesin on MT (PDB code: 4HNA (20)) were fitted into the cryo-EM map. **(D)** Focused 3D classification resulted in two distinct density maps showing either MAP7-bound (left) or kinesin-bound (right) tubulin, indicating competitive binding. **(E)** Fluorescent signal (top) and normalized intensity (bottom) of 1 μM LD655-labeled MAP7 constructs on undecorated, pre-decorated with 1 μM rigor kinesin or subtilisin-treated MTs (mean ±s.e.m., *n* values are given for each bar). **(F)** Kinesin motility in the presence of chimeric MAPs. **(G)** Run frequency and velocity of kinesin in the presence of Tau-MTBD/MAP7-C (mean ± s.e.m., *n* = 27, 112, 211, 102, 55 from left to right, two technical replicates).

Unexpectedly, kinesin run frequency decreased when MAP7 concentration was increased further (100-1,000 nM, Fig. 2D–E, movie S3). We reasoned that the nonlinear relationship between MAP7 decoration and kinesin motility could arise when the motor is subjected to simultaneous activation and inhibition that dominate at different concentrations (Fig. 2E) (19). To determine the source of these opposing inputs, we disrupted the kinesin-MAP7 interaction by truncating either the MAP7-binding domain of kinesin (K490, residues 1-490) or the kinesin-binding domain of MAP7 (MAP7-N and MAP7-MTBD, Fig. 3A). In all cases, MAP7 inhibited kinesin (Fig. 3B, fig. S8 and movies S5–6) as strongly as it inhibited dynein (Fig. 2E). Thus, MTBD inhibits while the projection domain activates kinesin (10).

MTBD may inhibit kinesin by competing for the same tubulin binding site, because superimposing the MT-bound structure of kinesin (20) onto our model reveals an apparent clash between kinesin and segment II of MAP7 (fig. S9A). Alternatively, the flexible segment II may accommodate kinesin binding by shifting away from the intra-dimer interface, as proposed for DCX and MAP4 (14, 21). To distinguish between these possibilities, we determined the structure of MTs incubated with rigor kinesin (K350^E236A^) and MAP7 (Fig. 3C). Because distinct binding sites of MAP7 and kinesin can be artifactually averaged during reconstruction (fig. S9B), we performed focused 3D classification around the putative shared binding site of MAP7/kinesin (fig. S10, see Methods). The classification resulted in two distinct maps of the binding site, one occupied by MAP7 only, and the other occupied by kinesin only (Fig. 3D). Thus, kinesin and MAP7 cannot simultaneously bind to the same tubulin dimer.

Consistent with cryo-EM, MTBD was unable to bind MTs pre-decorated with K350^E236A^ (Fig. 3E). However, FL MAP7 or a construct that lacks the MTBD (ΔMTBD, fig. S11A–C) still bound to kinesin-decorated MTs to a significant extent, presumably via the P123 domain (residues 175-316) (8, 9). MT binding of ΔMTBD was nearly abolished upon cleaving the flexible tails of tubulin (Fig. 3E, fig. S11D), explaining why P123 was invisible in our structure. To verify that kinesin is inhibited at high MAP7 decoration owing to binding site overlap, we also replaced the MT-binding regions of MAP7 with that of tau, which also overlaps with kinesin’s binding site and inhibits kinesin motility (fig. S12)(13, 22). Similar to FL MAP7, binding of this chimeric MAP to the MT resulted in biphasic regulation of kinesin motility (Fig. 3F–G).

**Fig. 4.**
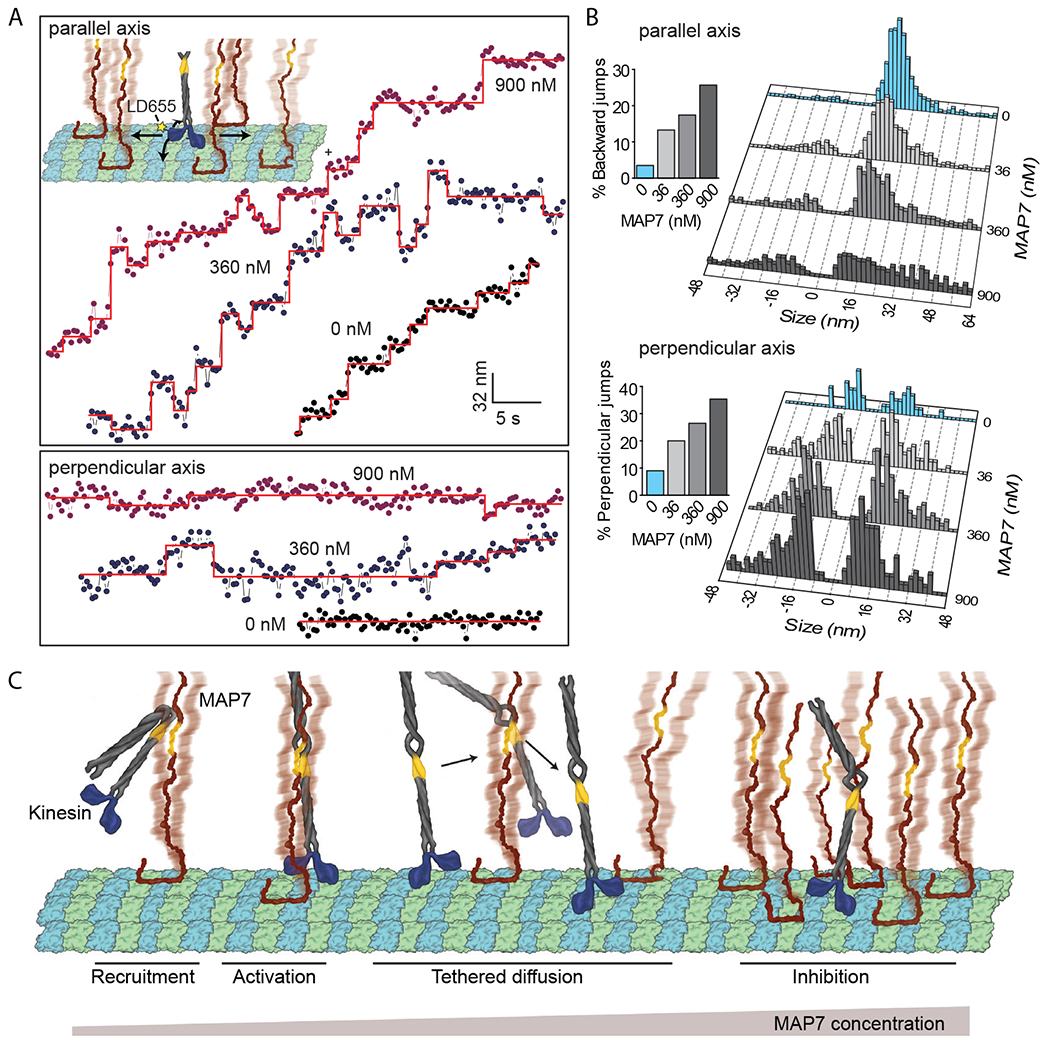
Kinesin bypasses MTBD obstacles via tethered diffusion. **(A)** (Insert) K560 was labeled with the LD655 dye at its N-terminus, and its stepping was tracked in longitudinal (straight arrows) and sideways (curved arrows) directions under different MAP7 concentrations. Representative traces of K560 motility along longitudinal (top) and sideways (bottom) directions. Horizontal lines represent a fit to a step-finding algorithm. **(B)** Histograms reveal the percentage of instantaneous jumps in backward and sideways directions under different MAP7 concentrations (bar graphs). From top to bottom, *n* = 437, 580, 502, and 331 for longitudinal and 43, 145, 195, and 181 for sideways directions. **(C)** Model for regulation of kinesin by MAPs. The MAP7 projection domain rescues kinesin from autoinhibition and tethers the motor to the MT. When kinesin encounters an MTBD obstacle, it dissociates from the MT, remains tethered to MAP7, and rebinds to the available tubulin site on another protofilament. Kinesin is inhibited at high MAP7 concentrations due to the scarcity of available binding sites.

To reveal how kinesin walks along MAP7-decorated MTs despite competing for the same binding site, we fluorescently labeled the motor domain of K560 and tracked kinesin stepping with nanometer precision under limiting ATP conditions (Fig. 4A). On undecorated MTs, kinesin took 16-nm steps in the forward direction (23), whereas steps in the sideways and backward directions were rare (9% and 3%, respectively; Fig. 4A–B, fig. S13A) (24). At 36 nM MAP7, we observed a modest increase in the probability of sideways and backward stepping (Fig. 4A–B). At nearly saturating MAP7 concentrations, we observed 16-64 nm displacements in our trajectories, as well as increased stepping in sideways (35%) and backward (26%) directions (Fig. 4A–B). Because kinesin cannot take such large steps on its own (Fig. 4A–B), these large displacements likely represent transient detachment and reattachment of the motor to the MT.

In the absence of the projection domain, kinesin was stuck on MTs decorated with 36 nM MTBD and dissociated from MTs at increased ATP concentrations (fig. S13B–C). Furthermore, the projection domain needed to be tethered to the MTBD to supersede kinesin inhibition, because the addition of the C-terminal half of MAP7 (MAP7-C) was unable to stimulate kinesin motility on MTs decorated with the N-terminal half of MAP7 (MAP7-N, fig. S14). Thus, the projection domain is essential for kinesin to bypass MTBD obstacles.

Based on our results and previous reports (9, 11), we propose a model for kinesin stepping on MAP7 decorated MTs. MAP7 recruits kinesin-1 to the MT and activates subsequent motility (9, 11). MT binding of MAP7 also obstructs kinesin stepping along the protofilament, which results in kinesin dissociation from the MT. However, the MAP7 projection domain tethers kinesin to the MT surface, allowing it to rebind the MT at nearby sites not blocked by its MTBD. This “tethered diffusion” of kinesin appears as large forward, sideways, or backward displacements in our trajectories. When the MT surface is nearly saturated with MAP7, the frequency and length of kinesin runs are reduced because of the scarcity of empty tubulin sites to which the motor can rebind after it dissociates (Fig. 4C). Unlike kinesin-1, MAP7 inhibits dynein motility because its MTBD overlaps with the dynein binding site (fig. S15) but its projection domain does not tether dynein to MTs.

This biphasic regulation mechanism may enable precise control of kinesin-1-driven transport by varying MAP7 density on cellular MTs. While MAP7 is required for kinesin-1 driven processes in many cell types (10, 25, 26), dense MAP7 localization at branch junctions in rat neurons slows down and pauses organelles at these sites (8). This mechanism may reroute cargos to their destinations by facilitating the detachment of kinesin from its MT track and its switching to neighboring MTs at cellular junctions (8).

## Supplementary Material

Ferro et al Supplement

Movie S1

Movie S2

Movie S3

Movie S4

Movie S5

Movie S6

Data S1
