# Supplementary material for: Structural and functional insight into the regulation of kinesin-1 by microtubule associated protein MAP7
Source: Science. Author manuscript; Available in PMC 2022 Apr 21. (PMC8985661; doi:10.1126/science.abf6154)
Supplement: Ferro et al Supplement [file NIHMS1786034-supplement-Ferro_et_al_Supplement.docx]

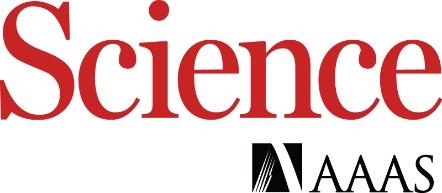


Supplementary Materials for

**Structural and functional insight into the regulation of kinesin-1 by microtubule associated protein MAP7**

Luke S Ferro, Qianglin Fang, Lisa Eshun-Wilson, Jonathan Fernandes, Amanda Jack, Daniel P Farrell, Mert Golcuk, Teun­­ Huijben, Katelyn Costa, Mert Gur, Frank DiMaio, Eva Nogales, Ahmet Yildiz

Correspondence to: [yildiz@berkeley.edu](mailto:yildiz@berkeley.edu), [enogales@lbl.gov](mailto:enogales@lbl.gov), qfang168@berkeley.edu

**This PDF file includes:**

Materials and Methods

Tables S1-S2

Figs. S1 to S16

Captions for Movies S1 to S6

Captions for Data S1

**Other Supplementary Materials for this manuscript include the following:**

Movies S1 to S6

Data S1

**Materials and Methods**

**Protein expression and purification**

Human MAP7 (UniProtKB Q14244-1), tau (UniProtKB P10636-8), and kinesin-1 (UniProtKB P33176-1) constructs have an N-terminal ZZ affinity tag, followed by a TEV protease cleavage sequence. MAP7 and tau constructs have an N-terminal ybbR sequence (DSLEFIASKLA) (*27*). Kinesin constructs have a C-terminal GFP and a SNAP-tag connected by a 1x GS linker sequence. The mouse BicDR1 (UniProtKB A0JNT9-1) construct has a C-terminal SNAP-tag. The BicDR1:Nanobody construct has a C-terminal GFP nanobody followed by the SNAP-tag. The phi mutant of the dynein-1 heavy chain (DHC; SNAP-DHC E1518K/R1567K) was co-expressed by fusing the coding sequence to the pDyn2 plasmid containing genes encoding IC2C, LIC2, TCTEX1, LC8, and ROBL1, as described (*28*). The list of constructs used in this study is shown in Table S1.

Protein purification was performed as previously described (*29, 30*). For kinesin constructs (FL kinesin, K560, and K490), a 500 mL cell pellet of Sf9 cells was thawed in 50 mL lysis buffer (25 mM HEPES pH 7.4, 1 M KCl, 10% glycerol, Roche protease inhibitor, 1 mM PMSF, 1 mM DTT, 0.1 mM ATP). The lysis was performed using a Wheaton glass dounce homogenizer. The lysate was spun for 45 min at 371,000 g (60k rpm) in a Ti70 rotor. The supernatant was incubated with 1 mL IgG sepharose beads for 1 h. Beads were then washed in 50 mL lysis buffer and 50 mL storage buffer (25 mM HEPES pH 7.4, 300 mM KCl, 1 mM EGTA, 10 mM MgCl_2_, 10% glycerol, 1 mM DTT, 0.1 mM ATP). Beads were resuspended with 1 mL storage buffer and transferred to a 2 mL Eppendorf tube. For SNAP-tag labeling, the bead-bound protein was incubated with 10 nmol SNAP-ligand conjugated dye for 1 h on ice. For ybbR-tag labeling, the bead-bound protein was incubated with coenzyme-A conjugated dye and 1 µM SFP enzyme for 30 min at room temperature. After labeling, beads were washed with 50 mL storage buffer. To elute the protein, beads were resuspended with 2 mL storage buffer and mixed with 30 µL of 2 mg mL^-1^ TEV protease for 30 min. Purification and labeling of the MAP7 constructs were identical to kinesin purification, except ATP was not added to the buffers. For dynein and BicDR, the same procedure was used using different lysis (50 mM HEPES pH 7.4, 100 mM NaCl, 10% glycerol, 1 mM DTT, 1 mM ATP, 2 mM PMSF, 1 Roche tablet per 50 mL) and storage (50 mM Tris pH 7.4, 150 mM KAc, 2 mM MgAc_2_, 1 mM EGTA, 10% glycerol, 1 mM ATP, 1 mM DTT) buffers.

The cysteine-light mutant (CLM) of human KIF5B (amino acids 1-560) K560-GFP-HaloTag CLM E215C was expressed in BL21DE3 cells and purified using Ni-NTA chromatography, as previously reported (*29*). K350^E236A^ expression plasmid was transformed into Rosetta2 (DE3) pLysS cells. Bacteria were grown in YT media until the culture reached an OD_600_ of 0.7. Cells were induced overnight at 20 ^o^C with 0.2 M IPTG, spun down at 4,785 g for 15 min in a JLA 8.1 rotor, and combined with 50 mL lysis buffer (50 mM NaH_2_PO_4_, 250 mM NaCl, 2 mM MgCl_2_, 30 mM imidazole, 10% glycerol, 1 mM DTT, 1 mM PMSF, pH 8.0). Cells were lysed by tip sonication and spun at 117,734 g in a Ti70 rotor. The supernatant was incubated with 1 mL of Ni-NTA beads for 1 h. Beads were washed with 50 mL wash buffer A (lysis buffer without PMSF), 50 mL wash buffer B (wash buffer A plus 750 mM additional NaCl) and exchanged back into wash buffer A. Protein was eluted with elution buffer (50 mM NaH_2_PO_4_ pH 7.2, 250 mM NaCl, 1 mM MgCl_2_, 500 mM imidazole, 10% glycerol). For SFP, the same procedure was used with different lysis (20 mM Tris, 0.5 M NaCl, 5 mM imidazole, pH 8) and elution (20 mM Tris, 0.5 M NaCl, 500 mM imidazole, pH 8) buffers. Protein concentrations were determined with either Bradford assay or absorbance at 280 nm on a Nanodrop spectrophotometer. Protein preps were run on an SDS-PAGE gel to check for purity and snap-frozen in liquid nitrogen for storage.

**Cryo-EM sample preparation**

Porcine brain tubulin (Cytoskeleton Cat # T240) was reconstituted to 10 mg/mL in BRB80 buffer (80 mM Pipes, pH 6.9, 1 mM ethylene glycol tetraacetic acid (EGTA), 1 mM MgCl_2_) with 10% (vol/vol) glycerol, 1 mM GTP, and 1 mM DTT. MTs were polymerized at 37°C for 20 min in BRB80 buffer supplemented with 266 μM peloruside (*31, 32*), pelleted and resuspended in the reaction buffer (MB buffer supplemented with 0.05% NP-40, 150 mM KAc, and 2 μM peloruside). Samples were incubated at a stoichiometry of 1:2 for MTs:FL-MAP7 and 1:3:10 for MTs:MAP7-MTBD:Tau for 5 min in the reaction buffer. MAP7 MTBD and FL tau were incubated with MTs in the absence of added salt. The MT concentration was set to 6 µM. The MAP/MT mixtures were added to glow-discharged C-flat holey carbon grids (CF-1.2/1.3–4C, 400 mesh, copper; Protochips) inside a Vitrobot (Thermo Fisher Scientific) set at 22 °C and 100% humidity, before plunge-freezing in ethane slush, and then grids were transferred to liquid nitrogen, as previously described (*13, 33, 34*).

The short MAP7 construct (MAP7^83-134^), FL MAP7, and rigor kinesin (K350, residues 1-350, E236A mutation) were desalted separately in the reaction buffer using Zeba Spin desalting columns (Pierce). For the short MAP7 construct dataset, the concentrations of tubulin and MAP7 were adjusted to 6 μM and 39 μM, respectively. Cryo-EM samples were prepared by incubating 2 µl of MTs on glow-discharged holey carbon grids (CF-1.2/1.3–4C, 400 mesh, copper; Protochips) for 30 s, adding 2 µl of MAP7 to the grid, and then incubating for 1 min. For the FL MAP7-kinesin dataset, the concentrations of tubulin, kinesin, and MAP7 were then adjusted to 6 μM, 14 μM, and 18 μM, respectively. Samples were prepared by first incubating 2 µl of MTs on glow-discharged holey carbon grids (CF-1.2/1.3–4C, 400 mesh, copper; Protochips) for 30 s, then adding 2 μl of kinesin for 30 seconds, and finally adding 2 μl of MAP7 for an additional 30 s. Sample vitrification was performed using a Vitrobot Mark IV (Thermo Fisher Scientific) at 22 °C and 100% humidity, with a blot force of 10 pN, blot time of 4s, and drain time of 1s.

**Cryo-EM data collection**

Data for MTs decorated with FL-MAP7, MAP7 MTBD, and MAP7-kinesin were collected using an Arctica microscope (Thermo Fisher Scientific), operated at an accelerating voltage of 200 kV (Table S2, fig. S16A-C). All cryo-EM images were recorded on a K3 direct electron detector (Gatan, Pleasanton, CA), at a nominal magnification of 36,000×, corresponding to a calibrated physical pixel size of 1.14 Å. The camera was operated in superresolution mode, with a dose rate of ∼16.2 electrons pixel^-1^ s^-1^ on the detector. We used an exposure time of ~4 s dose-fractionated into 50 frames, corresponding to a total dose of ~50 electrons Å^-2^ on the specimen.

Data for the short MAP7 truncation were collected using a Titan Krios G2 microscope (Thermo Fisher Scientific), operated at an accelerating voltage of 300 kV, equipped with a BIO Quantum Energy Filter (Gatan) (Table S2, fig. S16D). All cryo-EM images were recorded on a K3 direct electron detector (Gatan, Pleasanton, CA), at a nominal magnification of 64,000×, corresponding to a calibrated physical pixel size of 1.187 Å. The camera was operated in superresolution mode, with a dose rate of ∼15.7 electrons pixel^-1^ s^-1^ on the detector. We used an exposure time of ~4.5 s dose-fractionated into 50 frames, corresponding to a total dose of ~50 electrons Å^-2^ on the specimen. All the data were collected semiautomatically using the SerialEM software suite (*35*).

**Cryo-EM data processing**

The movie stacks were motion-corrected and dose-weighted using MotionCor2 (*36*), and the contrast transfer function parameters of each micrograph were estimated using gCTF (*37*). MTs were manually picked in RELION (*38*). MT segments along the length of each MT were extracted using a box size of 512 pixels, with neighboring boxes separated by 82 Å along the MT. The protofilament (PF) number of each MT was determined by performing supervised 3D classification using lowpass-filtered (20 Å) references comprising 13 and 14 PF MT as initial models, as previously reported (*39*). Particles from 13 PF MTs were selected for further processing. Global alignment parameters of the selected particles were determined in RELION using 20 Å-lowpass-filtered, 13-PF MT maps as initial models. The resulting global alignment parameters were input into FREALIGN (*40*) for further refinement. Helical parameters, determined using the *relion_helix_toolbox* function, were input to FREALIGN to impose pseudo-helical symmetry during reconstruction. The “good” PF was used to regenerate the full MT using EMAN2 libraries as previously described (*34, 41*). A previously described protocol was then used to distinguish αβ-tubulin and the seam location of each MT segment (*42*). The new alignment parameters were then refined using FREALIGN until convergence.

For the FL-MAP7 data set, the symmetry was expanded around the “good” PF of each MT segment using the previously determined helical parameters, thus enlarging the data set thirteenfold (fig. S10A). Focused 3D refinement using the cisTEM program (*43*) was then performed by masking the “good” PF until convergence. Masked signal subtraction (*44*) was then carried out to erase all but a single protofilament in each MT segment. Focused 3D refinement then gave the final map used for atomic modeling.

To determine if kinesin and MAP7 bind to the same tubulin dimer in the FL-MAP7-kinesin dataset, 3D classification with symmetry expansion and signal subtraction was performed (fig. S10B). First, the same symmetry expansion and signal subtraction procedure described above was carried out, resulting in the 3D reconstruction of a single PF. Second, another round of signal subtraction was performed by applying a soft mask around the PF in the 3D reference, followed by masking the density of one kinesin out. The signal subtracted particle images were then used to perform 3D classification in RELION with two classes by applying a soft mask around the kinesin density. This classification process gave one class (52.6%) showing density for kinesin but not for MAP7, and another class (47.4%) showing density for MAP7 but not for kinesin. To show the density map in the context of complete tubulin densities, we reconstructed the final maps of the kinesin class and MAP7 class using the particle images before signal subtraction making use of the class number assignment information from the focused classification.

**Atomic modeling**

The final map for the MT sample bound to FL-MAP7 was used for atomic modeling (Table S2). The map was initially interpreted by fitting tubulin structures from a previous report (PDB ID: 6o2r) (*33*) into the tubulin density, and by building a Cα model that contains a 53-residue-long α helix into the MAP7 density in Coot (*45*). Because the local resolution of MAP7 was not sufficient for unambiguous sequence assignment (fig. S1B), sequence registration determination was carried out in Rosetta using a method similar to previous work (*13*). Since the map using FL-MAP7 and that using MAP7-MTBD show the same density ascribed to MAP7, only the MAP7-MTBD sequence (residues 60-170) was considered for sequence registration determination, giving 59 possible sequence registrations to consider. The Rosetta program *partial_thread* was used to thread the Cα model of MAP7 at all possible sequence registrations. Each threaded MAP7 model, combined with the models of a tubulin dimer and an α-tubulin contacting with MAP7, was then subjected to refinement against the cryo-EM density using RosettaCM (*15, 46*). For each of the 59 starting models, 100 refined models were generated. The lowest-energy four of these were subjected to further refinement against the cryo-EM density using the *relax* application in Rosetta (*47*), where 20 models were generated from each of these starting points. Finally, for each threading, the five lowest-energy models were selected for further analysis.

While proteins that decorate the MT lattice shorter than the length of a tubulin dimer (~82 Å) can be fully resolved using our image analysis methodology, those that are longer would be averaged over part of their length because the molecules would not be in register from one protofilament to the next (fig. S4A). Such regions of averaged density would be uninterpretable in terms of secondary structure (fig. S4B). The secondary structure prediction showed α-helical tendency for additional N-terminal (residues 64-86) and C-terminal (residues 140-150) MAP7 regions that would potentially extend the modeled helix on both ends. Focused 3D classification around the MAP7 densities did not reveal a class with a larger helix (not shown), but this approach may have failed due to the relatively low signal of a single alpha helical segment. Inspection of our density map showed no obvious blurring effect of the helical backbone, indicating that if the MAP7 helix on the MT surface were to be larger than currently modelled, the extended part must be coincident on the C_α_ atoms in the averaged regions. Indeed, a hypothetical model that includes the additional N- and C- terminal portions of the MAP7 helix (residues 64-150; fig. S4B), shows that residues that interact with tubulin within the 118-140 segment share high sequence similarity (fig. S4B) with the corresponding residues 64-86 that are expected to interact with equivalent tubulin regions.

**Fluorescence microscopy**

Fluorescence microscopy was performed using a Ti-E Eclipse inverted microscope body equipped with a 100x 1.49 NA Apo TIRF objective (Nikon). 488, 532, and 633 nm laser beams (Coherent) were fiber-coupled using laser-to-fiber couplers and a wave dimension multiplexer (OZ Optics). TIRF illumination was controlled using a TI-TIRF Motorized Illuminator unit (Nikon). The emission signal was filtered using 512/25, 580/40 697/75 nm bandpass filters (Chroma) mounted in a Lambda 10-B optical filter changer (Sutter). Images were collected with an electron-multiplied CCD camera (Andor, Ixon^+^). The microscope was controlled using Micromanager. The effective pixel size after magnification was 160 nm.

**Motility assays**

Tubulin was purified from pig brains as described (*48*) and labeled with biotin or fluorophores. The final percentage of labeled tubulin in the MTs was less than 5%. Coverslips coated with PEG/PEG-Biotin (Microsurfaces) were assembled into 10 µL flow chambers with laser-cut Parafilm. All solutions for motility assays were in MB buffer (30 mM HEPES, 5 mM MgSO_4_, 1 mM EGTA, pH 7.0). 1 mg/mL streptavidin was added to the chamber and incubated for 2 min. Following three washes with 30 µL wash buffer (MB buffer supplemented with 0.5% Pluronic F-127, 1 mg/mL casein, 1 mM TCEP, 10 µM taxol), MTs were added to the chamber and allowed to attach for 2 min. Unattached MTs were removed by an extensive wash of the flow chamber and the chamber was exchanged into the imaging buffer (wash buffer supplemented with 150 mM KAc, 0.1% methylcellulose, 1 mM ATP, glucose oxidase, catalase, 0.4% glucose, motors, and MAPs). The sample was sealed and imaged immediately. Reported MAP concentrations represent the concentration of MAP added to the imaging buffer.

As reported previously (*17*), FL kinesin was autoinhibited and exhibited only occasional motility in the absence of MAP7 (fig. S7). K560 and K490 are constitutively active dimers and they exhibit robust motility even in the absence of MAP7 (Fig. 3B). While all three constructs moved at similar velocities (~800 nm s^-1^) in 2 mM ATP, K490 had ~6-fold higher run frequency than K560 in the no MAP condition (fig. S8C) (*9*). The motility of all three constructs was slowed down by an increase in MAP7 concentration, but under the same MAP concentrations (100 nM), the velocities of FL kinesin (100 nM FL MAP7), K560 (100 nM MAP7-N), and K490 (100 nM FL MAP7) decreased by 37%, 50%, and 10%, respectively. This difference could be related to negative interactions between kinesin’s stalk (between residues 490-560) with the MT, as previously proposed (*9*).

Kinesin purified from insect cells has similar velocity, but longer run lengths (6 µm on average) than kinesin expressed in *E.coli* (2 µm) under the same assay conditions (*49*). Experiments in Figure 2D-E were conducted using K560 expressed in *E. coli* for a direct comparison between our results and the results of the previous publications using the same construct (*9, 10*). In comparison, experiments in Figure 3B and figure S8C were collected using K560 expressed in insect cells. The differences between run lengths of kinesin expressed in *E. coli* vs. insect cells do not impact our conclusions, as we also observed biphasic regulation of FL kinesin expressed in insect cells by MAP7 (fig. S7), similar to K560 expressed in *E.coli* (Figure 2D-E). See Table S1 for the constructs and their expression system used for each figure panel.

Fitting was performed in OriginPro 9, Prism 9, and MATLAB 2016a. MT binding curves of fluorescently-labeled MAPs were fit to a Langmuir binding isotherm: $I (\left[ MAP \right])=\frac{I_{max}[MAP]}{k_{d}+\left[ MAP \right]}$, where *I* is the fluorescence intensity of a MAP on the MT, *k_d_* is the half-maximal saturation concentration and *I_max_* is the fluorescence intensity of a MAP at saturation. The run frequencies of motors under different MAP concentrations were fitted to the *Hill1* equation in Origin with the Hill coefficient set to 1: $RF(\left[ MAP \right])=MIN+\frac{MAX-MIN}{1+\frac{[MAP]}{{IC}_{50}}}$, where *RF* is the normalized run frequency, *MIN* is the minimum value, *MAX* is the maximum value, and *IC_50_* is the half-maximal inhibition. The only exception was the run frequency of K560 on FL MAP7, which was fitted to *BiHill* equation in Origin, where Hill coefficients were set to 1: $RF(\left[ MAP \right])=\frac{{RF}_{max}}{\left( 1+\frac{K_{a}}{[MAP7]} \right)\left( 1+\frac{[MAP7]}{K_{i}} \right)}$, where *RF_max_* is the maximum run frequency, *K_a_* is the half-maximum activating MAP7 concentration, *K_i_* is the half-maximal inhibiting MAP7 concentration. Run frequency values were normalized to 1 at 0 nM MAP conditions.

**KD assemblies**

The dynein complex (0.6 µM) was labeled with SNAP-LD655 (Lumidyne Technologies) on an N-terminal SNAP-tag on the dynein heavy chain and mixed with unlabeled dynactin (1 µM), and the BicDR1 construct (1 µM), which contains a C-terminal GFP-nanobody. After incubating the mixture on ice for 15 min, FL kinesin-1-GFP-SNAP labeled with LD555 (0.5 µM) was added and the mixture was incubated on ice for another 10 min. Biotinylated-MTs were attached to PEG-Biotin coverslips. Wash buffer included Pluronic and casein. The kinesin-dynein complex was diluted 500-fold in imaging buffer (wash buffer supplemented with 1 mM ATP, glucose oxidase, catalase, 0.4% glucose, and MAPs).

Movies were acquired using time-sharing between 532 and 633 nM laser exposures (200 ms each). Files were analyzed using Bio-Formats (ImageJ). In the “no MAP7” condition, immotile or diffusive spots in the dynein channel were attributed to dynein-only motors. Stationary molecules were filtered from the kymograph in the Fourier space using a fast Fourier transform (FFT) mask in ImageJ. Two fluorescent channels were registered to identify trajectories that contain both fluorescent dyes. Velocities of these trajectories were measured by determining the distance between binding and dissociation and the time over which the motors stepped, including pauses and backtracking events.

**Stepping analysis**

To track kinesin stepping, Cys-lite human kinesin-1 with a single surface-exposed cysteine (K560 CLM E215C) was expressed in Rosetta2 (DE3) pLysS cells and purified as described above. Protein was then labeled with 2x molar excess maleimide-conjugated LD655 (Lumidyne) at 4 ℃ overnight, and free dye was removed using a 40kDa Zeba spin desalting column (Thermo Fisher). Imaging chambers were coated with biotinylated MTs as described above. Imaging buffer containing K560 and varying concentrations of MAP7 and ATP was flown into the chamber. Unbound motors were washed off, and the imaging chamber was sealed with nail polish. Movies were collected at 3 Hz for 90 s. To reliably detect >90% of the steps taken by motors at 3 Hz temporal resolution, motility was slowed down to ~5 nm s^-1^ by adjusting the ATP concentration under different MAP7 conditions (15, 15, 100, and 1,000 nM ATP for 0, 36, 360, and 900 nM MAP7, respectively). The diffraction-limited spots of individual motors were localized using a two-dimensional Gaussian fitting tracker algorithm, and the resulting traces were fit to steps in MATLAB 2017a using a Schwartz information criterion algorithm, as previously described (*50, 51*). Fitted traces were visually inspected and incorrectly identified steps were removed manually.

**MD simulations**

The atomic model of MAP7 (residues V87-H139) on α-β-α tubulins without C-terminal tails (Fig. 1C) were solvated in a water box with padding of at least 15 Å of water in each direction using the TIP3P water model. Systems were ionized to 150 mM KCl. The MAP7-tubulin system was composed of ~140,000 atoms. MD simulations were run in NAMD 2.14 (*52*) using the CHARMM36 all-atom additive protein force field (*53*). Simulations were run using 2 fs time step, 310 K temperature, 1 atm pressure. The particle-mesh Ewald method was used to calculate long-range electrostatic interactions, and 12 Å cut-off distance was used for van der Waals interactions. The protein was fixed for 10,000 steps of minimization followed by 1 ns of equilibration. Subsequently, constraints on the protein were released and the system was minimized for an additional 10,000 steps, followed by 2 ns of equilibration applying Harmonic potential with a spring constant of 1 kcal mol^−1^Å^−2^ to the C_α_ atoms. The production run was initiated following these two minimization cycles. This procedure was repeated 7 times to obtain extensive conformational sampling. To account for the missing MAP7 and MT structure, C_α_ atom positions of tubulin residues P325-R339 (first α tubulin) and E71-G81 (second α tubulin) and MAP7 residues V87 and H139 were constrained with a spring constant of 0.5 kcal mol^−1^Å^−2^. Starting from different sample conformations, two 200 ns long MD simulations and two 150 ns long simulations were performed. Root-mean-squared fluctuations of the C_α_ atoms of MAP7 residues were calculated compared to initial conformation. The formation of salt bridges between the basic nitrogen and acidic oxygens was determined with a 6 Å cutoff distance (*54*). To detect hydrogen bonding, a 3.5 Å cutoff distance with a 30° cutoff angle was used between the hydrogen atom, the donor heavy atom, and the acceptor heavy atom (*55*).

**Supplementary figures**

**
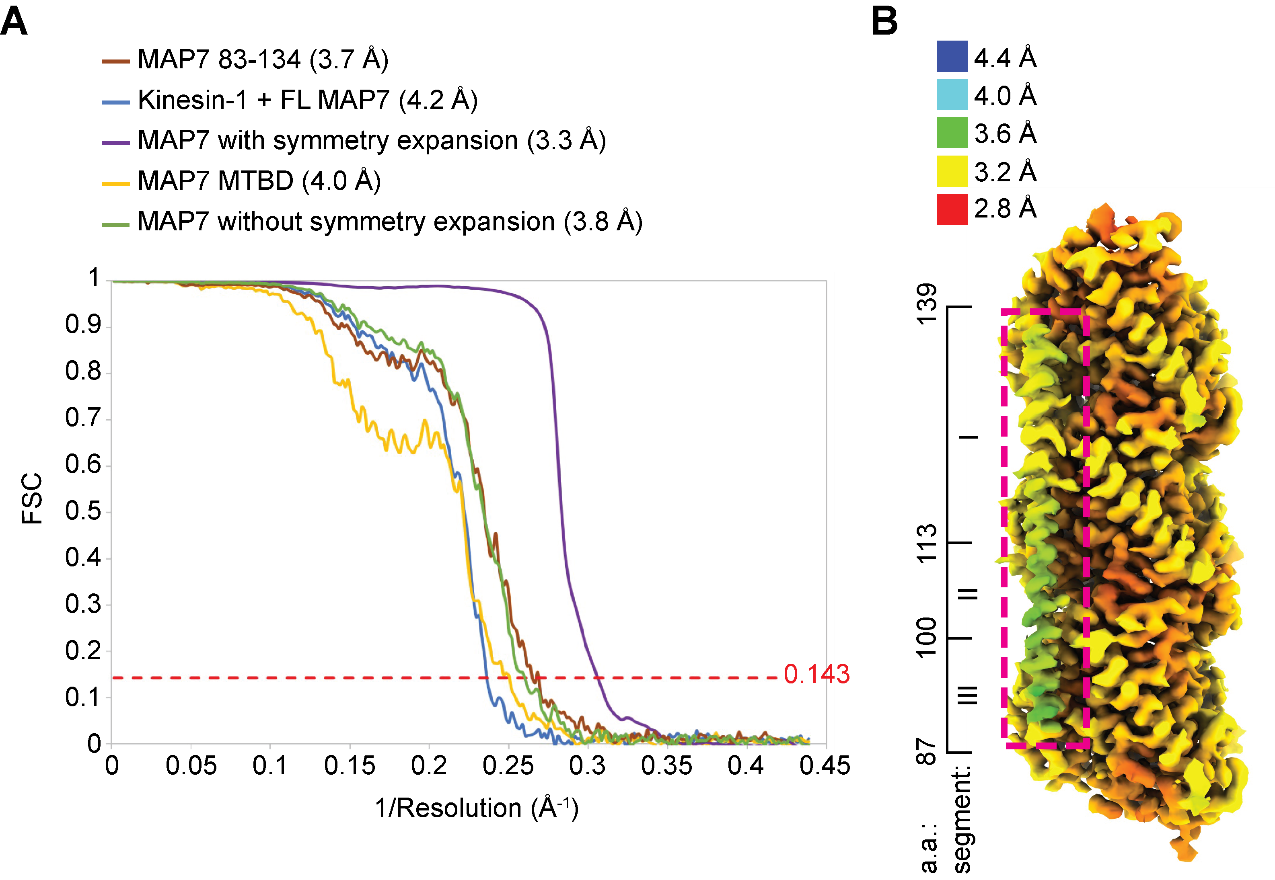
**

**Fig. S1. Resolution estimations of the cryo-EM maps. (A)** Fourier Shell Correlation (FSC) plots of the cryo-EM maps reveal the final resolution based on the gold-standard criterion (FSC = 0.143). **(B)** Local resolution of the final map (after symmetry expansion and protofilament-based density subtraction) of an MT decorated with MAP7 as calculated using BSoft (*56*) with a 0.5 FSC cutoff.

**
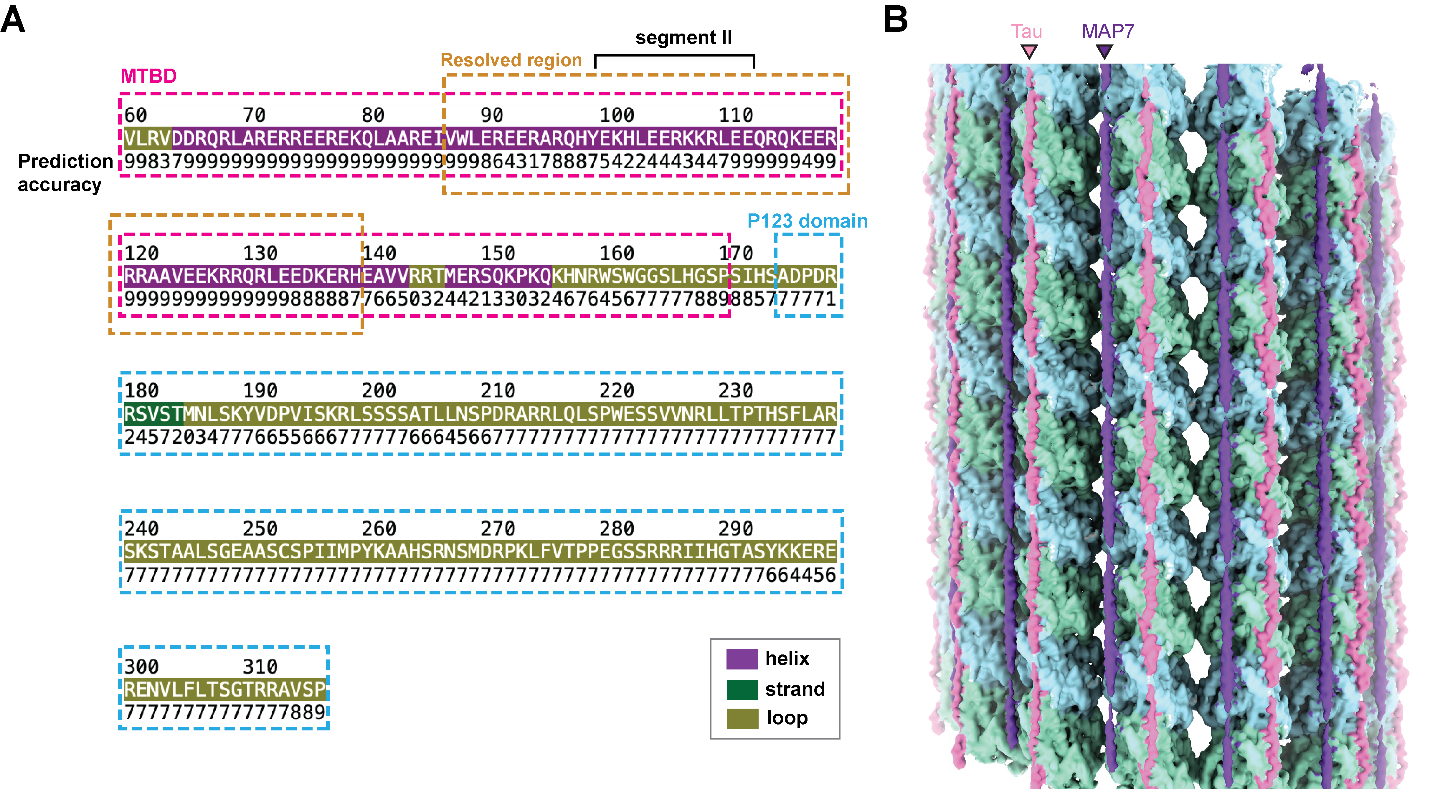
**

**Fig. S2.** **Secondary structure prediction and co-structure of MAP7 MTBD and FL tau. (A)** Secondary structure prediction for the MAP7 MTBD and P domain**,** calculated using JPred (*57*). The numbers indicate confidence level (9 being the highest score). **(B)** Cryo-EM map of an MT decorated by MAP7 MTBD (residues 60-170) and FL tau. The α-tubulin, β-tubulin, MAP7, and tau are shown in green, blue, purple, and pink, respectively.

**
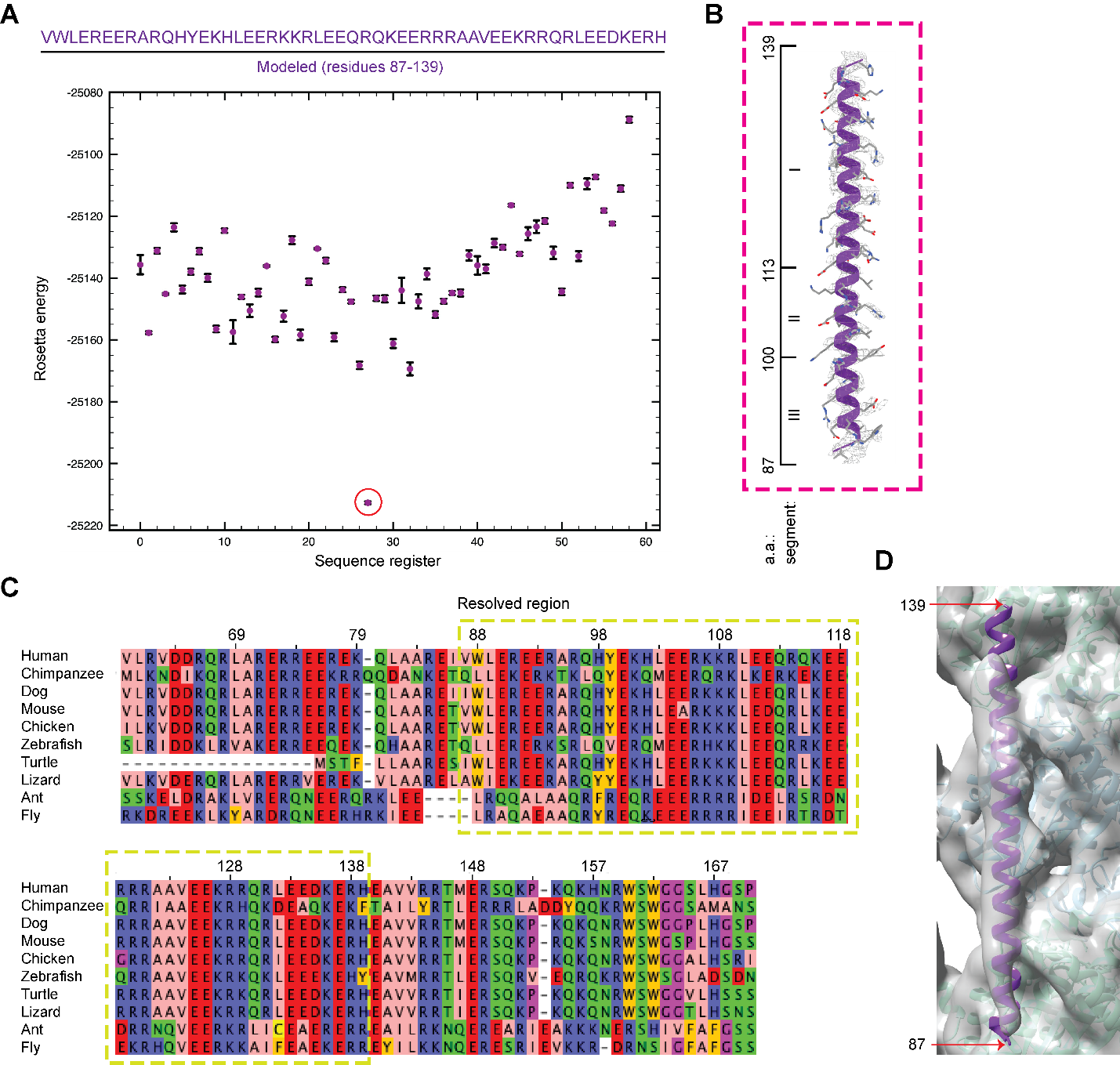
**

**Fig. S3. Sequence registration of the cryo-EM map of MAP7.** **(A)** Rosetta modeling support one sequence register energetically. **(B)** Model of MAP7 (ribbon with side chains) on the MAP7-MT cryo-EM map after symmetry expansion and protofilament-based density subtraction. The cryo-EM map is shown as mesh. **(C)** Sequence alignment of the MAP7 MTBD from different species. Positively charged residues, negatively charged residues, and polar residues are colored blue, red, and green, respectively. Aromatic residues and cysteine residues are colored orange and yellow, respectively. Hydrophobic residues are colored light pink. Proline and glycine are colored purple. **(D)** Cryo-EM structure of MTs in complex with a short MAP7 truncation (lowpass-filtered to 8 Å) containing only residues: 83-134 (MAP7^83-134^). The atomic model of the MT decorated by FL-MAP7 is fitted into the density map using UCSF Chimera to show the missing density of MAP7.


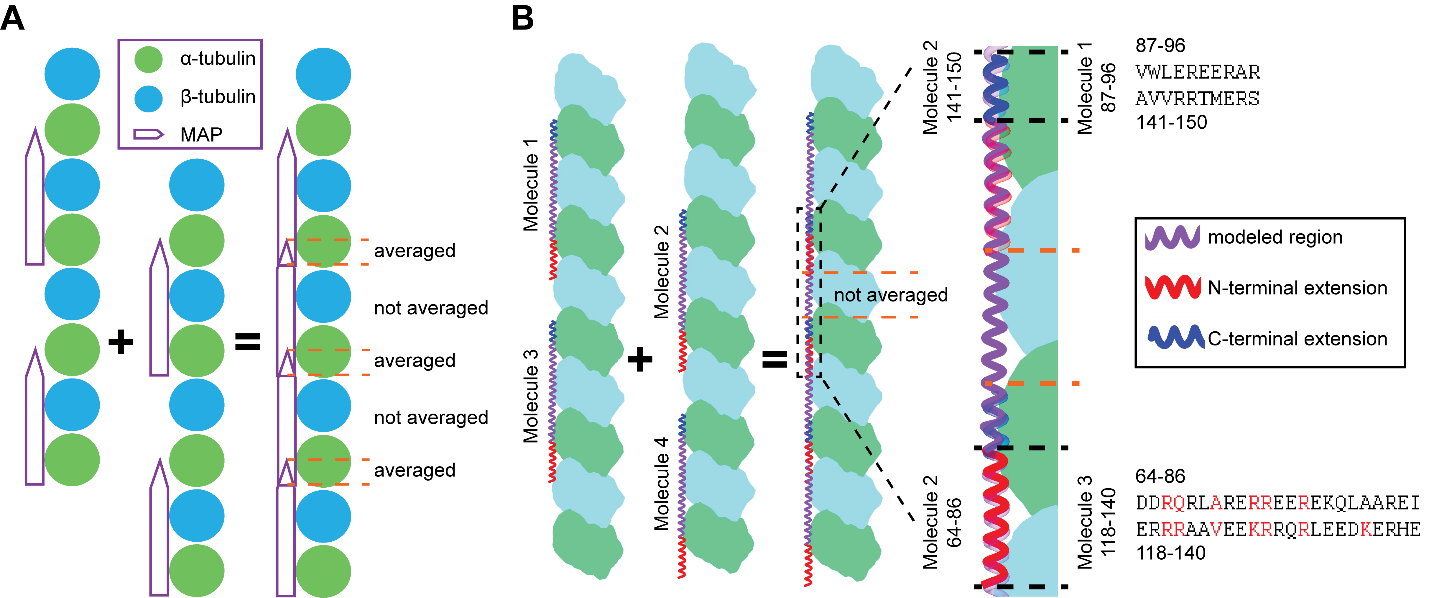


**Fig. S4. Possible extension of the MAP7 footprint on the MT. (A)** If the MAP binding site along the protofilament were longer than the length of a tubulin dimer, it would result in averaging of the overlapping regions during cryo-EM reconstruction. **(B)** (Left) A hypothetical extended model of the MAP7 helix (residues 64-150) highlights the possible overlapping regions on the MT. (Right) The sequence alignment for the regions of an extended helix and those that would superimpose with it in the present atomic model. Residues that contact the MT in 118-140 and 64-86 segments are marked in red. α- and β-tubulin are shown in green and blue, respectively.

**
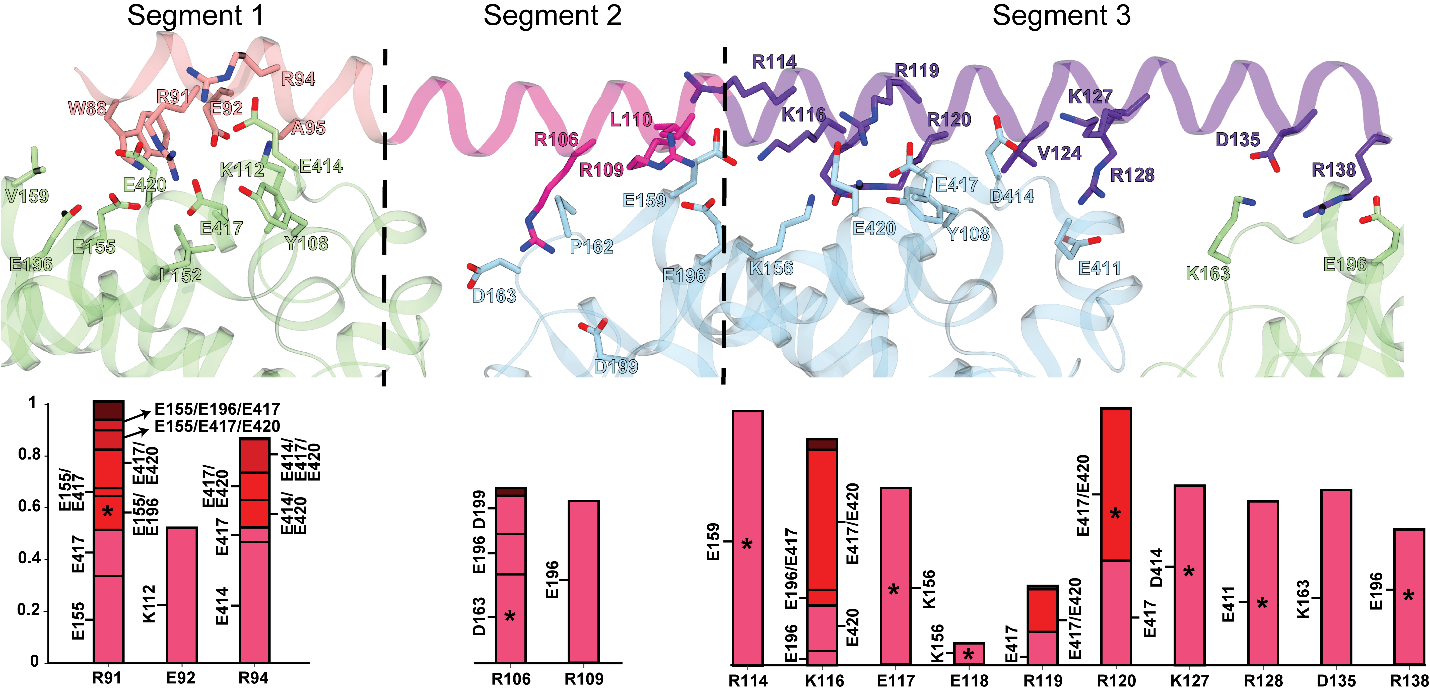
**

**Fig. S5. Dynamic interactions between MAP7 and tubulin residues observed in all-atom MD simulations.** (Top) Interactions between MAP7 segments I-III and tubulin. α tubulin and β tubulin are shown in green and blue colors. (Bottom) The frequency of observed interactions between MAP7 and tubulin for in total of 700 ns long MD simulations. MAP7 residues are shown on the x-axis whereas the tubulin residues are indicated in the bars. Crimson, red, and maroon colors indicate single, double, and triple interaction pairs of MAP7 residues with tubulin. Mahogany denotes the frequency of other interactions observed less than 3% of the simulation time.


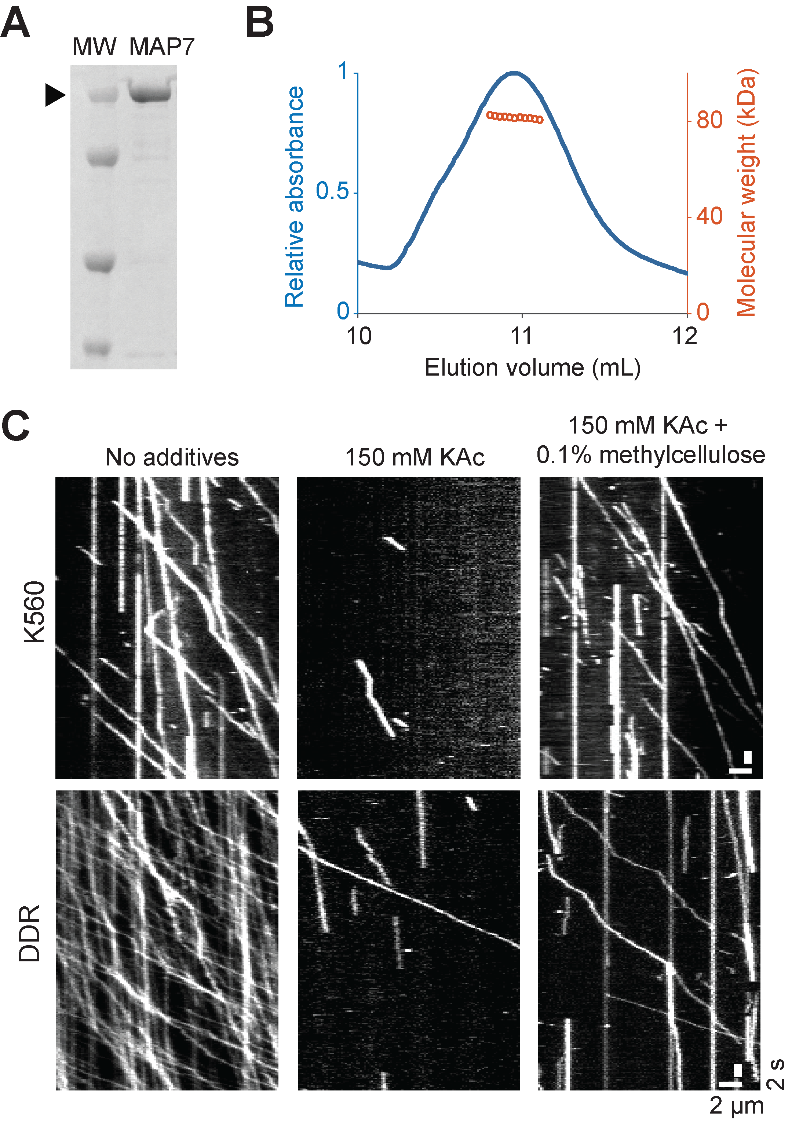


**Fig. S6. MAP7 is a monomer in the solution. (A)** Denaturing gel of purified MAP7 (MW: molecular weight). **(B)** Size exclusion chromatography coupled to the multi-angle light scattering (SEC-MALS) shows MAP7 elutes as a monomer. **(C)** The run frequency and run length of K560 and DDR substantially decrease under physiological salt (150 mM KAc), but the addition of a crowding agent (0.1% methylcellulose) rescues the motility in these conditions.


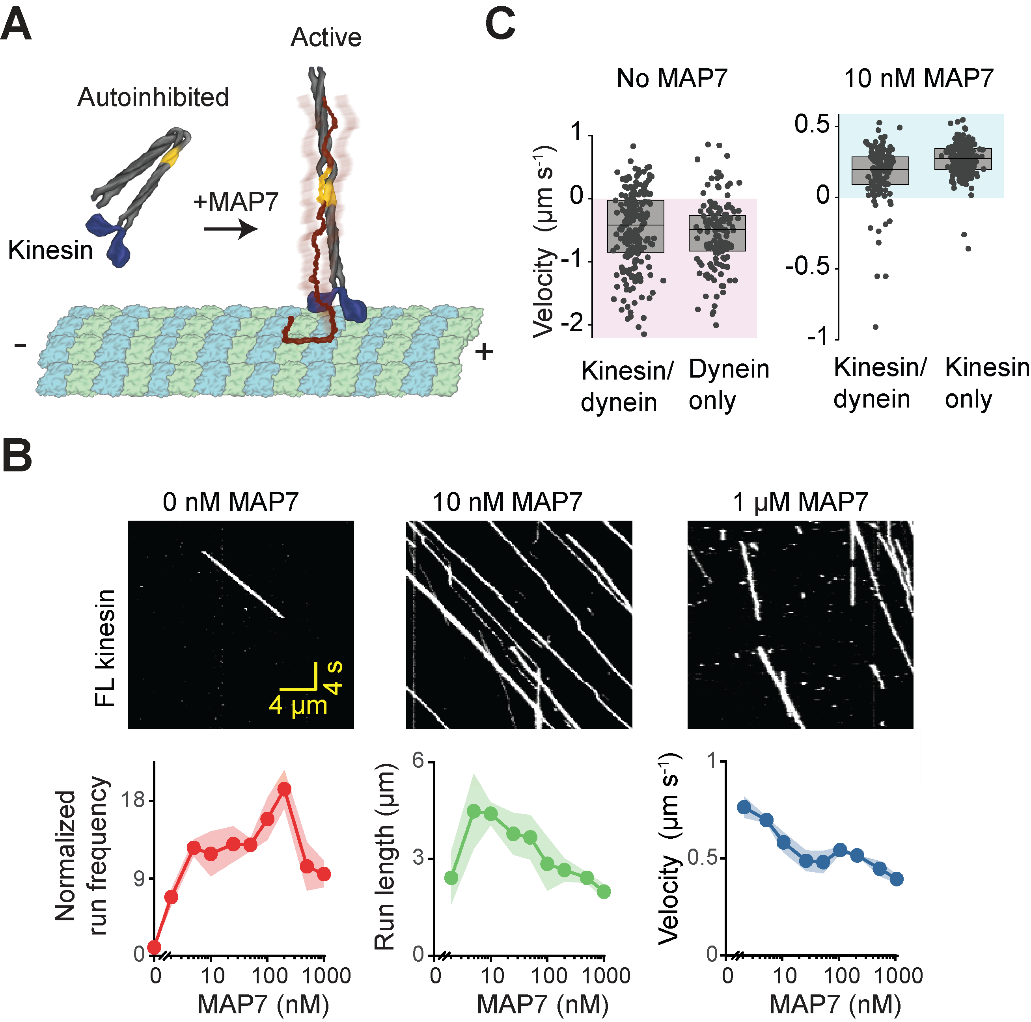


**Fig. S7. Biphasic regulation of FL kinesin by MAP7. (A)** Schematic shows the activation of FL kinesin by MAP7 on the MT. **(B)** (Top) Kymographs of FL kinesin motility with and without MAP7. (Bottom) The run frequency, run length, and velocity of FL kinesin at different MAP7 concentrations. FL kinesin exhibits only occasional motility in the absence of MAP7. The addition of 10 nM MAP7 activates its motility and increases its run frequency and run length (*n* = 12, 658, 399, 527, 816, 664, 2434, 1303, 897, 802 runs from left to right; two technical replicates). **(C)** The velocity analysis of kinesin-dynein assemblies in the absence and presence of 50 nM MAP7. (Left) The velocity of complexes that contain both LD655-dynein and LD555-kinesin versus LD655-dynein only in the absence of MAP7. N = 207, 151 from left to right. (Right) The velocity of complexes that contain both LD655-dynein and LD555-kinesin versus LD555-kinesin only in 10 nM MAP7. N = 145, 435 from left to right. Experiments were repeated 3 times without additional KAc or methylcellulose.


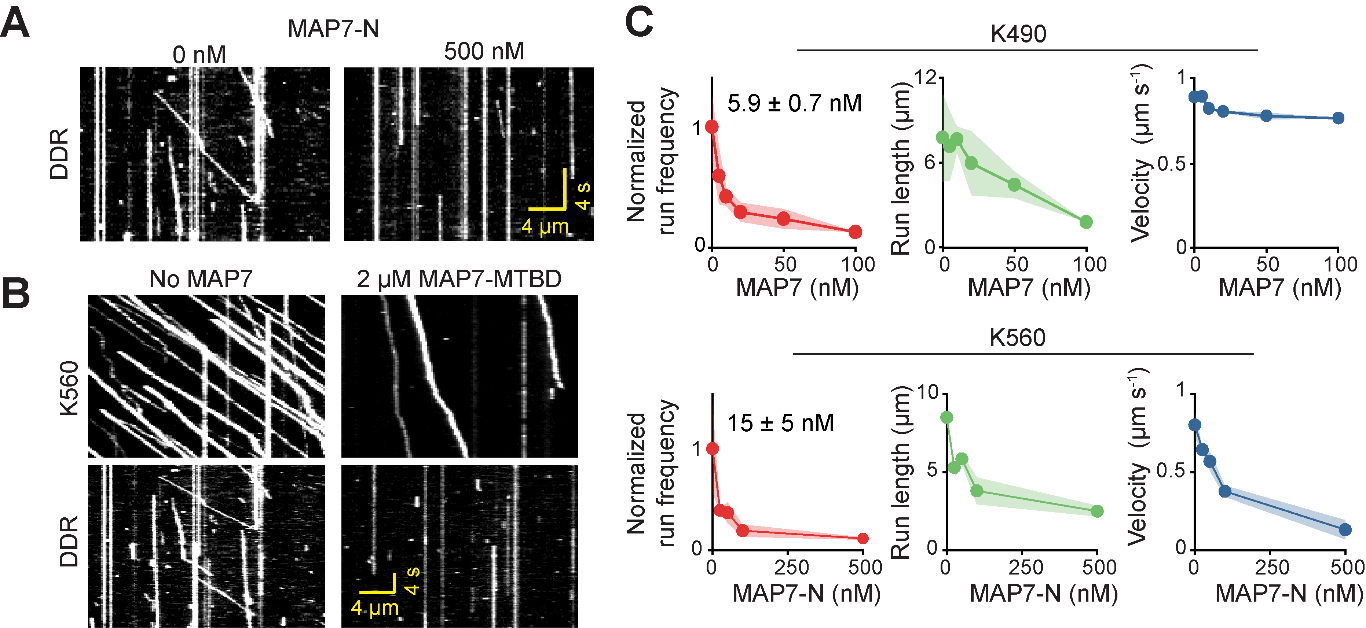


**Fig. S8. Kinesin motility is inhibited by the MT binding of MAP7 without the MAP7 projection domain. (A)** Kymographs show that MAP7-N inhibits DDR motility. **(B)** Kymographs show that MAP7-MTBD inhibits K560 and DDR motility. **(C)** Run frequency, run length, and velocity of kinesin constructs under different MAP7 concentrations. Error bars represent s.d. IC_50_ (±s.e.) of run frequency was calculated from a fit to Langmuir equation (From left to right, *n* = 855, 532, 581, 304, 457, 149 for K490 and 276, 168, 172, 121, 75 for K560; two technical replicates). P values are calculated from a two-tailed t-test.


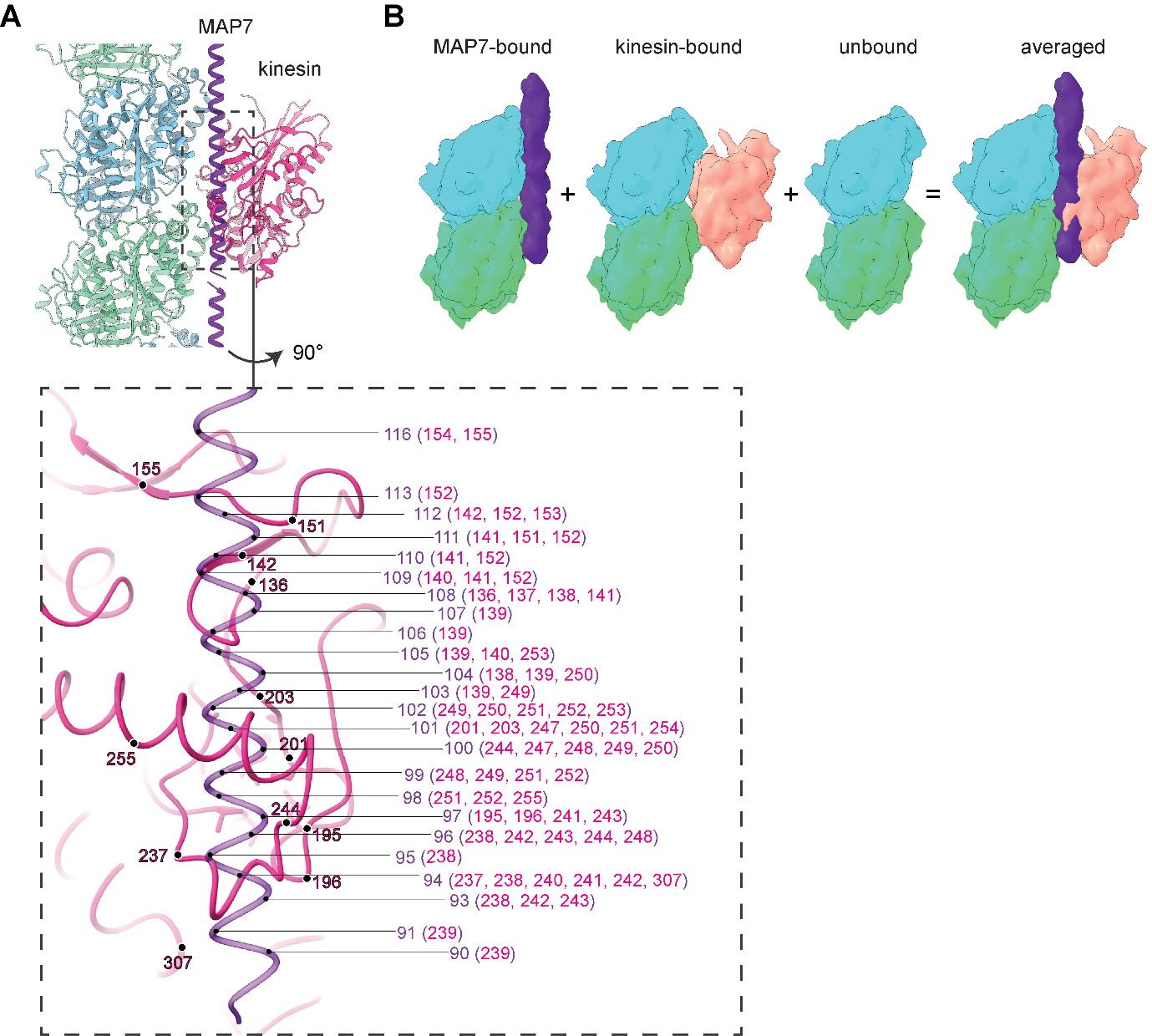


**Fig. S9. MAP7 competes with kinesin for binding to the MT.** **(A)** (Top) The kinesin-1 motor domain was placed in the MAP7-MT model by superimposing the tubulin part of our MAP7-MT model onto that of a previously reported structure of Kinesin-MT (PDB code: 4HNA). (Bottom) Details of the clashes between MAP7 (purple) and kinesin-1 (pink) upon MT binding. Atoms from one MAP7 residue separated less than 2.2 Å from any atom of a kinesin residue are considered to clash with each other. The tubulin models are not shown for clarity. **(B)** Diagram showing how the map in Figure 3C could be generated by averaging different binding states along the MT lattice.

**
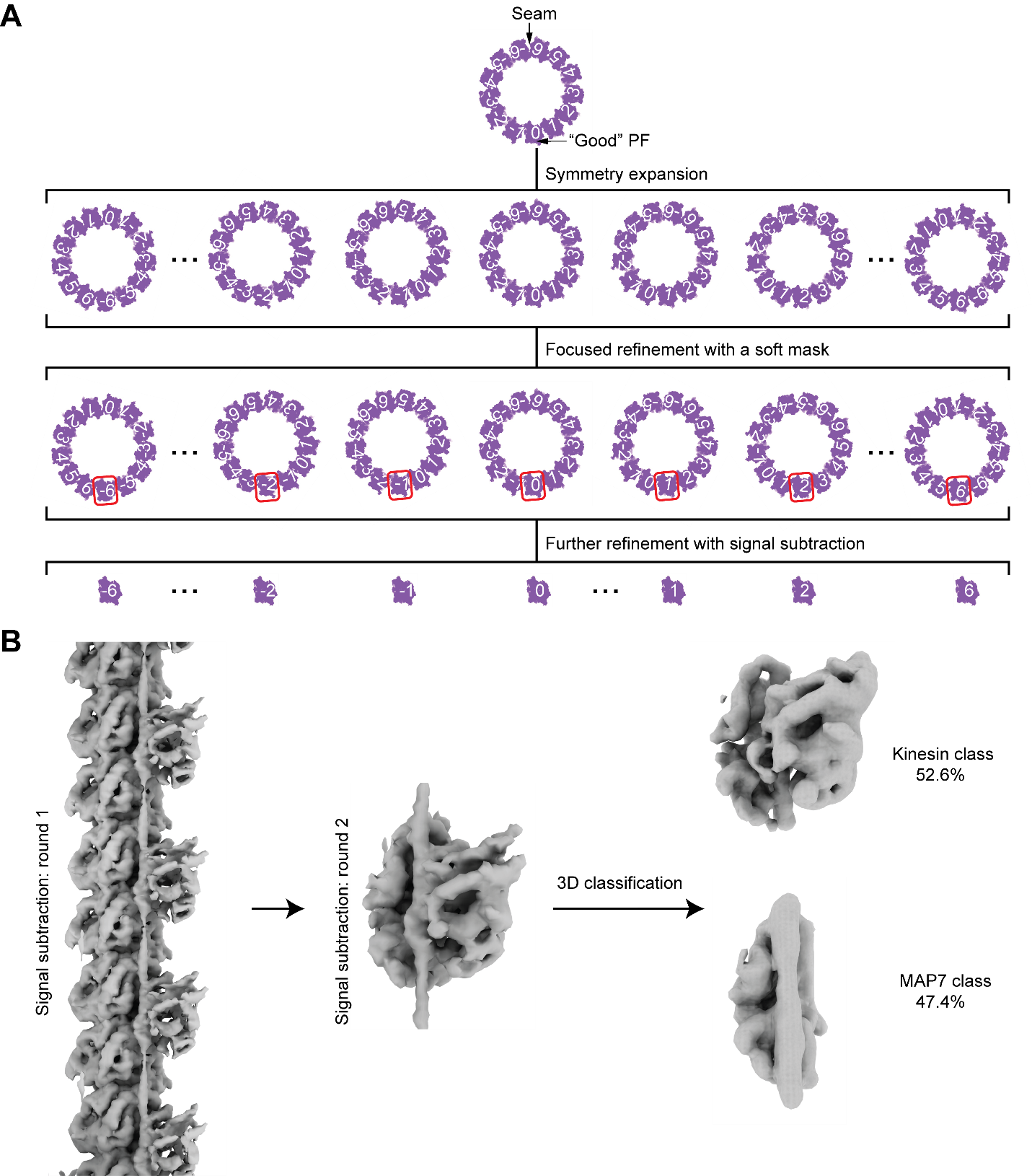
**

**Fig. S10. Cryo-EM data processing scheme for focused refinement of the FL-MAP7 data set (A) and 3D classification of the MAP7-Kinesin data set (B).**


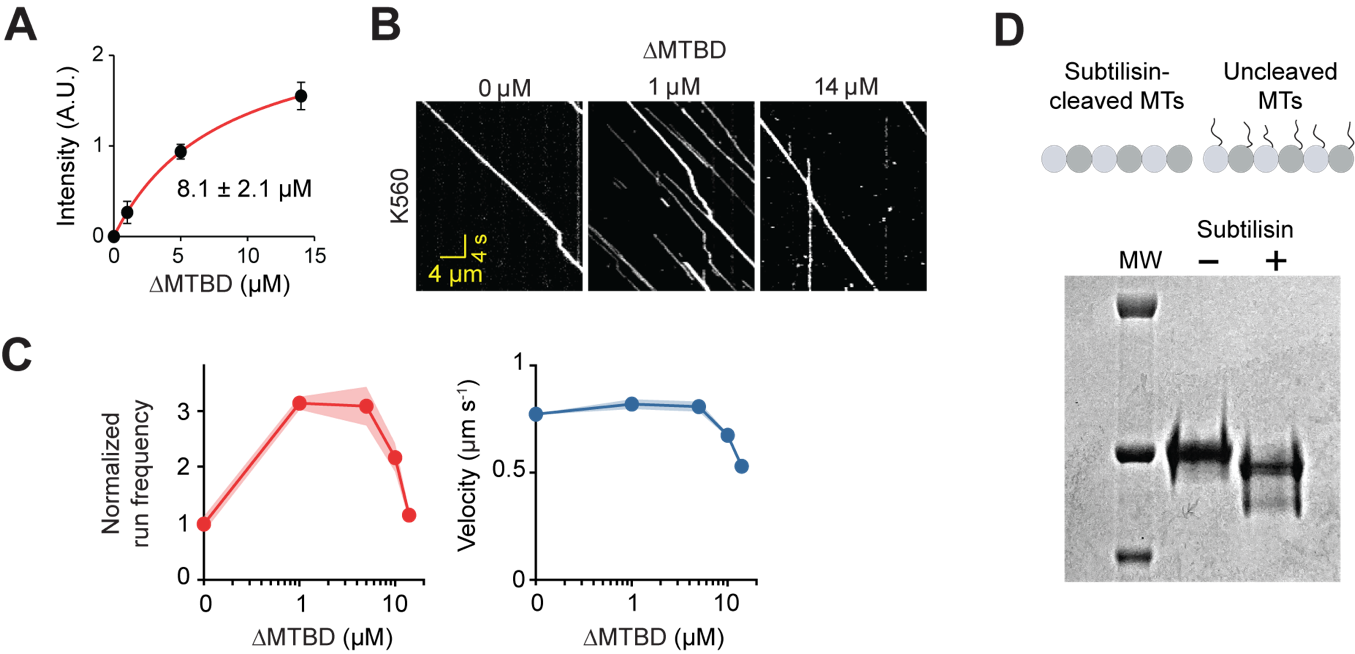


**Fig. S11. MAP7 binds to MTs and regulates kinesin motility in the absence of its MTBD.** **(A)** Fluorescent intensity of LD655- ΔMTBD MAP7 on surface-immobilized MTs (mean ± s.e.m.). The solid curve represents a fit to a Langmuir binding isotherm to calculate K_d_ (± s.e.; *n* = 20, 24, 20, 26 MTs; two technical replicates). **(B)** Representative kymographs of K560 under different ΔMTBD concentrations. **(C)** Run frequency and velocity of K560 under different ΔMTBD concentrations (mean ± s.e.m.). The run frequency exhibits biphasic behavior by increasing ΔMTBD concentrations, presumably due to negative interactions between kinesin and ΔMTBD on the MT surface (*n* = 35, 143, 129, 116, 51 from left to right; two technical replicates). **(D)** (Top) Schematic of subtilisin cleavage of the C-terminal tails of tubulin. (Bottom) The denaturing gel shows the reduction of the molecular weight (MW) of tubulin upon subtilisin treatment.

**
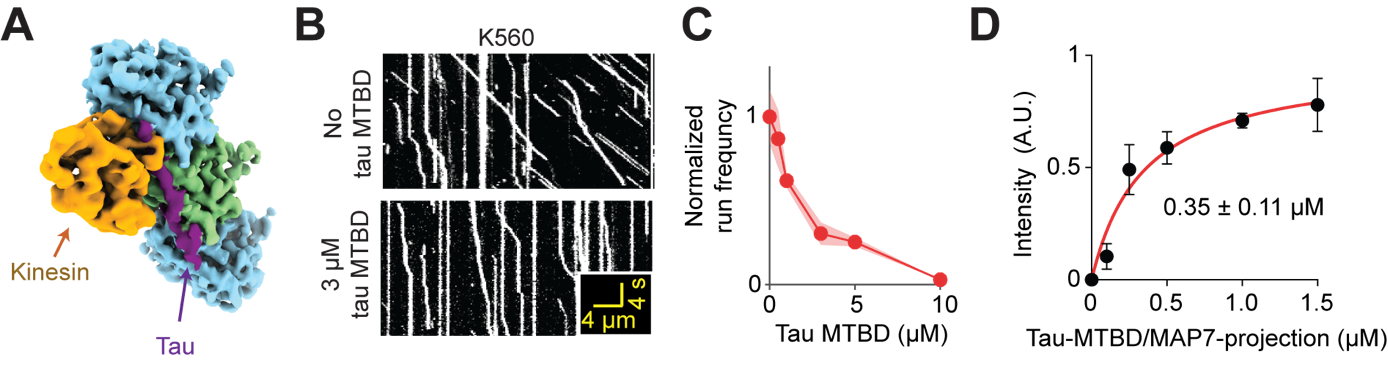
**

**Fig. S12. The MTBD of tau overlaps with kinesin’s binding site and inhibits kinesin motility.** **(A)** The density of the kinesin motor domain (EMDB ID: 6353) was superimposed on the density of tau (EMDB ID: 7769) bound to the MT. **(B)** Representative kymographs of kinesin walking in the presence and absence of 3 μM tau MTBD. **(C)** Run frequency of kinesin motors in the presence of an increasing concentration of tau MTBD. Fit to a Langmuir equation (not shown) reveals IC­_50_ = 1.8 ± 0.5 μM (*n* = 848, 801, 839, 568, 499, 46 from left to right; two technical replicates). **(D)** TIRF binding assay of tau MTBD- MAP7-C construct to the MT. Fit to a Langmuir binding isotherm (red curve) reveals K_d_ (*n* = 22, 31, 21, 19, 21 MTs from left to right; two technical replicates).


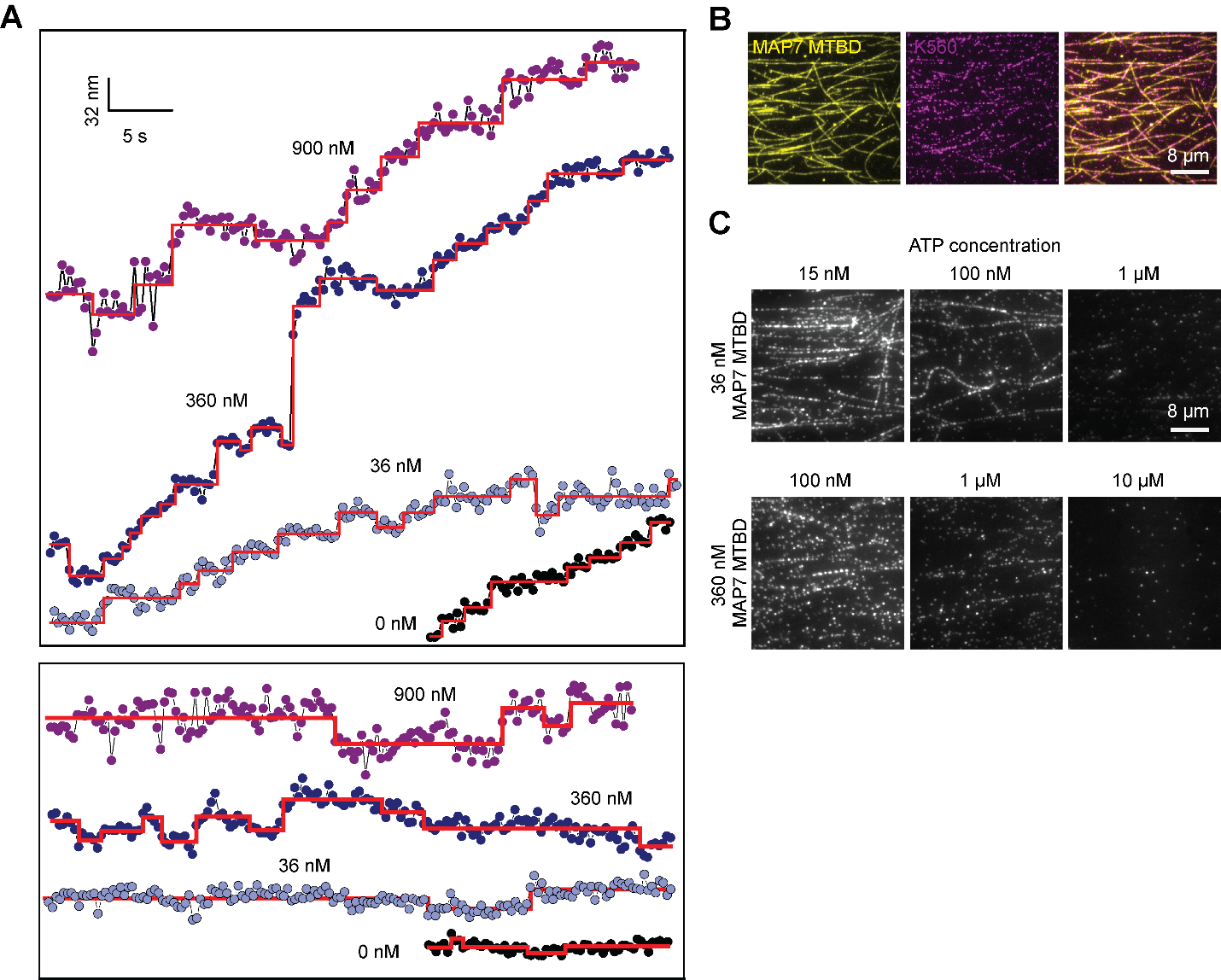


**Fig. S13. Kinesin stepping on MAP7-decorated MTs. (A)** Additional example trajectories of K560 stepping in the presence or absence of MAP7. Symbols represent the position of the LD655 dye attached to the K560 N-terminus along longitudinal (top) and sideways (bottom) directions relative to the MT axis. Horizontal lines represent a fit to a step-finding algorithm. **(B)** 360 nM MAP7 MTBD and K560 colocalize on MTs in the presence of 100 nM ATP. However, MT decoration of K560 at desired linear density required a 100-fold higher concentration than in the FL MAP7 stepping experiments. **(C)** K560 localizes to the MT but does not walk in the presence of MAP7 MTBD and low ATP. Increasing ATP concentration reduces the number of kinesin proteins bound to the MTs decorated by MAP7 MTBD, indicating that kinesin stalls at MTBD obstacles and dissociates from the MT by hydrolyzing ATP.

**
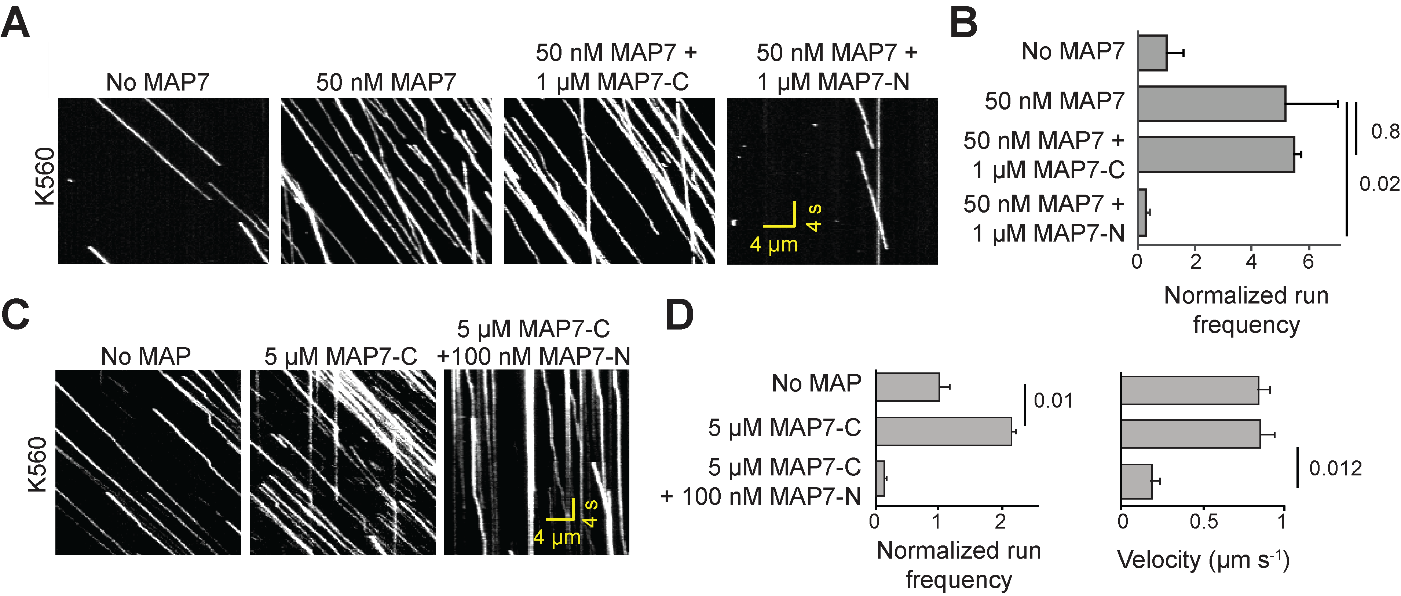
**

**Fig. S14. The projection domain of MAP7 must be tethered to MTBD to enable kinesin motility. (A)** Kymographs of K560 motility in the presence of MAP7 construct combinations. **(B)** K560 run frequency for different MAP7 construct combinations. From top to bottom, N = 114, 355, 365, 27 runs. Error bars represent s.d. p-values are calculated from a two-tailed t-test. Assays were performed in 150 mM KAc and 0.1% methylcellulose. **(C)** Kymographs of kinesin motility in the presence of MAP7-C or both MAP7-C and MAP7-N. **(D)** Run frequency and velocity of kinesin in the presence of MAP7-C or both MAP7-C and MAP7-N (mean ± s.e.m.; *n* = 152, 447, 18 from top to bottom; two technical replicates). P values are calculated from a two-tailed t-test.

**
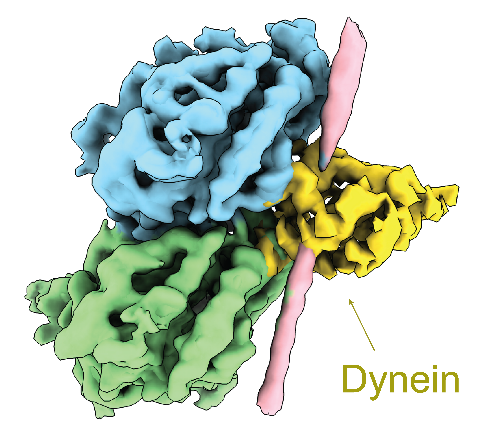
**

**Fig. S15. The MAP7 MTBD overlaps with the dynein binding site on tubulin.** The density of the dynein-MTBD (EMDB ID: 10060) was superimposed on the MAP7 density on the MT.

**
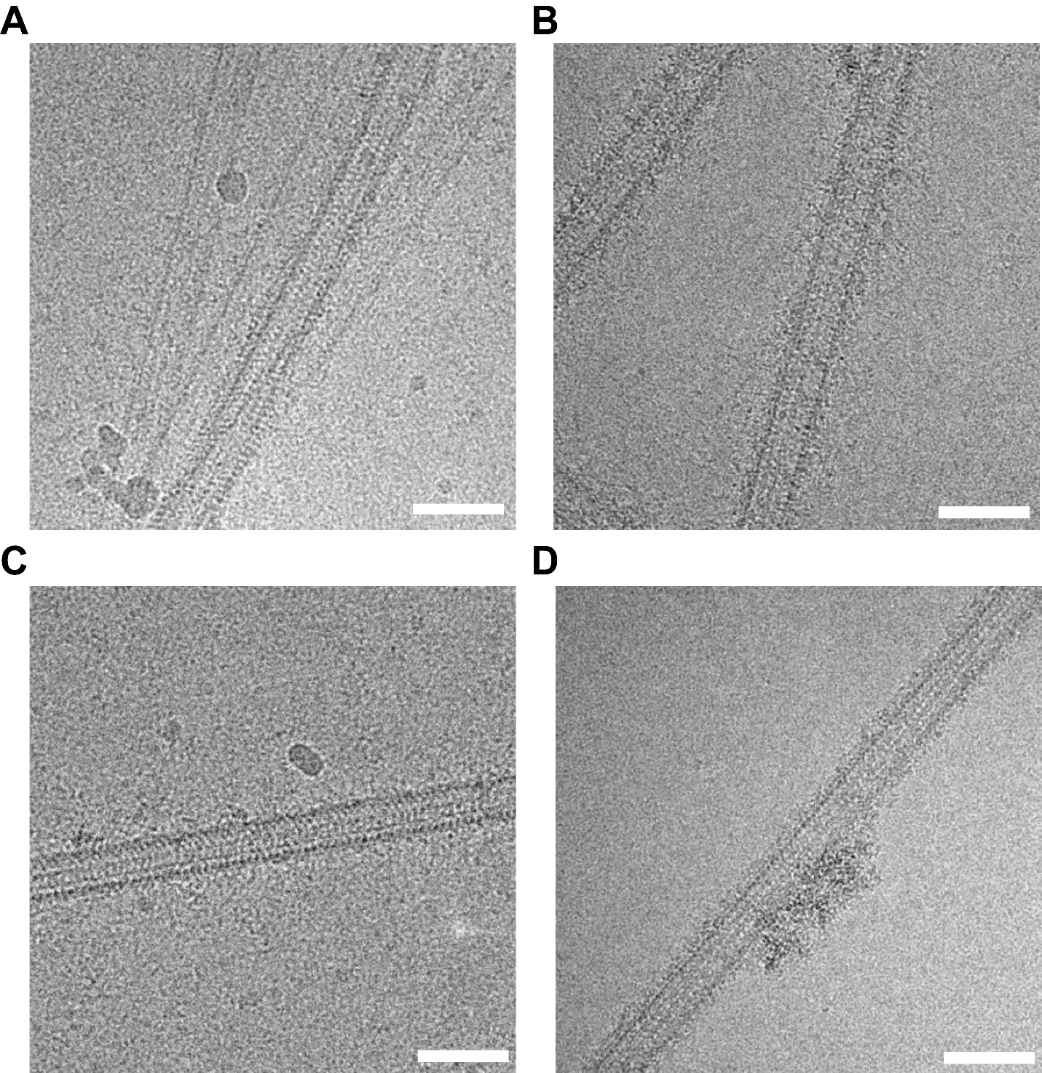
**

**Fig. S16. Representative cryo-EM image of the MAP7-MTBD dataset (A), MAP7-kinesin dataset (B), FL-MAP7 dataset (C), and MAP7^83-134^ dataset (D).** The scale bars represent 50 nm.

**Supplementary Tables**

| **Name** | **Description** | **Source** | **Expression** | **Figures** |
| --- | --- | --- | --- | --- |
| K490 | Human KIF5B^1-490^ ::GFP:: SNAP | This study | Sf9 | 3B, S8C |
| K560 | Human KIF5B^1-560^ ::GFP:: SNAP | This study | Sf9 | 3B, S8B-C |
| K560-GFP E215C CLM | Human KIF5B^1-560 E215C^ ::GFP::HaloTag (Cys-light) | Ferro et al. 2019 (*29, 30*) | E. coli | 2D-E, 4, S6, S13 |
| FL KIF5B | Human KIF5B^1-963^ ::GFP:: SNAP | This study | Sf9 | 2F-H, S7 |
| K350^E236A^ | Human KIF5B^1-350 E236A^ | This study | E. coli | 3C-D, S9 |
| FL MAP7 | Human ybbR::MAP7^1-749^ | This study | Sf9 | 1A-D, 2B-H |
| MAP7-N | Human ybbR::MAP7^1-316^ | This study | Sf9 | 3B, S14 |
| MAP7 MTBD | Human ybbR::MAP7^60-170^ | This study | Sf9 | 3E, S8B, S13 |
| MAP7^83-134^ | Human MAP7^83-134^ | This study | E. coli | S3D |
| MAP7-C | Human ybbR::MAP7^307-749^ | This study | Sf9 | S14 |
| ΔMTBD | Human ybbR::MAP7^171-749^ | This study | Sf9 | 3E, S11 |
| MAP7-MTBD/  tau-projection | Human ybbR::tau^1-241^-MAP7^60-170^-tau^366-441^ | This study | Sf9 | 3F-G |
| FL Tau | Human ybbR::tau^1-441^ | This study | Sf9 | S3D |
| Tau-MTBD/  MAP7-projection | Human ybbR::tau^191-417^-MAP7^307-749^ | This study | Sf9 | 3F-G |
| Tau MTBD | Human ybbR::Tau^242-367^ | This study | Sf9 | S2, S12 |
| SNAPf-Dyn-Phi | SNAPf-DYNH1C1^K1610/R1567E^-IC2C-LIC2-Robl1-Tctex1-LC8 | Schlager et al. 2014 (*28*) | Sf9 | 2D-H |
| BicDR1-SNAP | Mouse BicDR1_1-577_ ::SNAP | This study | Sf9 | 2D-E |
| BicDR1-SNAP-GFP | Mouse BicDR1_1-577_ ::SNAP::GFP nanobody | This study | Sf9 | 2F-H |

**Table S1. The list of constructs used in this study.** Amino acid numbers and points mutations are shown in subscripts and superscripts respectively.

| Name | MAP7 FL | MAP7 MTBD | MAP7^83-134^ | K350 + FL MAP7 |
| --- | --- | --- | --- | --- |
| PDB ID | 7SGS | N/A | N/A | N/A |
| EMDB ID | 25120 | 25119 | 25117 | 25118 |
| Microscope | Arctica | Arctica | Krios | Arctica |
| Voltage (kV) | 200 | 200 | 300 | 200 |
| Camera | K3 | K3 | K3 | K3 |
| Defocus range (µm) | 1.2-2.5 | 1.2-2.5 | 1.2-2.5 | 1.2-2.5 |
| Automation software | SerialEM | SerialEM | SerialEM | SerialEM |
| Exposure time (s) | ~4 | ~4 | ~4.5 | ~4 |
| Dose rate (electrons/pixel/s) | ~16.2 | ~16.2 | ~15.7 | ~16.2 |
| Frames | 50 | 50 | 50 | 50 |
| Total dose (electrons/Å^2^) | 50 | 50 | 50 | 50 |
| Pixel size (Å/pixel) | 1.14 | 1.14 | 1.187 | 1.14 |
| Number of micrographs | 1,792 | 2,631 | 3,176 | 5,718 |
| Starting number of particles | 135,535 | 50,223 | 37,977 | 142,414 |
| Number of particles (final map) | 99,219 | 37,442 | 23,162 | 55,873 |
| Map sharpening B factor (Å^2^) | -60 | N/A | N/A | N/A |
| Map sharpening methods | RELION | N/A | N/A | N/A |
| Symmetry | C1 | Helical | Helical | Helical |
| Overall resolution (Å) | 3.3 | 4.0 | 3.7 | 4.2 |
| Resolution range of MAPs (Å) | 3.2-4.0 | 4.0-6.5 | 5.5-8.5 | 4.5-8.5 |

| Initial model used (PDB ID) | 6O2R |
| --- | --- |
| Refinement package | Rosetta |
| C-beta outliers | 0.00 |
| Rotamer outliers (%) | 0.78 |
| All-atom Clashscore | 0.56 |
| MolProbity score | 0.69 |
| Ramachandran plot (outliers / favored) (%) | 0.07 / 98.59 |
| Ligand | 5 |
| Protein residues | 1357 |
| R.m.s.d. of bond lengths (Å) | 0.020 |
| R.m.s.d. of bond angles (°) | 1.630 |
| CC (mask) | 0.81 |
| CC (volume) | 0.77 |
| EMRinger score | 3.75 |

**Table S2. Cryo-EM data collection parameters and model refinement statistics.** Cryo-EM data collection parameters (top) and model refinement statistics of MAP7 FL (bottom) (r.m.s.d.: root-mean-squared deviation).

**Movie captions****Movie S1. Cryo-EM structure of MAP7 on the MT generated using UCSF ChimeraX.**

**Movie S2. Interactions between MAP7 and tubulin in MD simulations.** A representative all-atom MD simulation of our atomic model shows dynamic interactions between MAP7 residues and tubulin.

**Movie S3. K560 and DDR motility on MTs in the presence of different MAP7 concentrations.** K560 motors were labeled with LD555-BG on a C-terminal SNAP-tag. The DDR complex was labeled with LD555-BG on a C-terminal SNAP-tag of BicDR-1. MAP7 is added at the given concentration along with the motor solution and is not washed out from the chamber. Motility buffer includes 150 mM KAc, 0.1% methylcellulose, glucose oxidase, catalase, dextrose, and 1 mM ATP. K560 and DDR motility were recorded at 5 Hz under TIRF illumination. In a separate fluorescent channel (not shown), MT decoration on the PEG-biotin coverslip was recorded.

**Movie S4. Motility of kinesin-DDR assemblies in the presence and absence of MAP7.** FL kinesin with a C-terminal GFP was labeled with LD555-BG on a C-terminal SNAP-tag. BicDR, with a C-terminal anti-GFP nanobody, was expressed in Sf9 cells. Dynein was purified from Sf9 cells and fluorescently labeled with LD655 on an N-terminal SNAP-tag. DDR complex was assembled on ice and mixed with kinesin to form kinesin-DDR assemblies. Two-color movies were collected under TIRF illumination. Each channel’s exposure time was 200 ms. The motility of assemblies is shown in the presence of 10 nM MAP7. Motility buffer includes glucose oxidase, catalase, dextrose, and 1 mM ATP.

**Movie S5. K490 motility on MTs in the presence of different MAP7 concentrations.** K490 was labeled with LD555-BG on a C-terminal SNAP-tag. In a separate fluorescent channel (not shown), MT decoration on the PEG-biotin coverslip was recorded. Kinesin motility is shown in the presence of 0, 10, 100 nM MAP7 in the chamber. Motors and MAP7 were flown into the chamber and images were collected at 5 Hz under TIRF illumination. Motility is shown in the presence of 0, 5, 75 nM MAP7. Motility buffer includes 150 mM KAc, 0.1% methylcellulose, glucose oxidase, catalase, dextrose, and 1 mM ATP.

**Movie S6. K560 motility on MTs in the presence of different MAP7-N concentrations.** K560 motors were labeled with LD555-BG on a C-terminal SNAP-tag, and their motility was recorded at 5 Hz under TIRF illumination. In a separate fluorescent channel (not shown), MT decoration on the PEG-biotin coverslip was recorded. Kinesin motility is shown in the presence of 0, 50, 100 nM MAP7-N in the chamber. Motility buffer includes 150 mM KAc, 0.1% methylcellulose, glucose oxidase, catalase, dextrose, and 1 mM ATP.

**Supplementary Data Captions**

**Data S1. The list of all individual quantitative observations that underlie the data summarized in the figures of this study.** Multiple panels of each figure were included as separate sheets in a single Excel file.
